# A novel ene-reductase from *Halomonas elongata* for flow biocatalytic synthesis of 3-phenylpropionaldehyde and sustainable indigo-carmine dyeing

**DOI:** 10.1039/d5ra06869j

**Published:** 2025-11-12

**Authors:** Lauriane Pillet, Cristina Lía Fernández Reguerio, Markus Richard Busch, David Roura Padrosa, Francesca Paradisi

**Affiliations:** a Department of Chemistry, Biochemistry and Pharmaceutical Sciences, University of Bern Freiestrasse 3 CH-3012 Bern Switzerland francesca.paradisi@unibe.ch; b InSEIT AG Freiestrasse 3 CH-3012 Bern Switzerland

## Abstract

In order to broaden the toolbox of enzymes available for biocatalytic reductions of carbon-carbon double bonds, we investigated four promising ene-reductases (ERs) stemming from extremophilic organisms or showing homology with thermophilic ERs. The novel ene-reductase from the halophilic organism *Halomonas elongata* showed consistently high activity across a range of tested substrates. Upon immobilisation of the ERs, the flow biocatalytic ene-reduction of cinnamaldhehyde into 3-phenylpropionaldehyde was successfully achieved with an intensification of the process of 62-fold with respect to batch (2173.9 mg L^−1^ h^−1^ and 34.7 mg L^−1^ h^−1^, respectively). Additionally, to expand the scope of ERs applications, we describe a proof-of concept of a novel enzymatic cascade to convert indole to indigo using an unspecific peroxygenase, and its subsequent reduction to the water-soluble leuco-indigo. In addition, the co-production of the valuable pharmaceuticals precursor 2-oxindole was demonstrated.

## Abstract 2

The characterization of novel ene-reductases identifies a halophilic enzyme with remarkable catalytic efficiency. *He*OYE could be successfully immobilised for process intensification and applied in a tandem cascade for the synthesis of leuco-indigo. The image was created in full using Bria AI.

## Graphical abstract

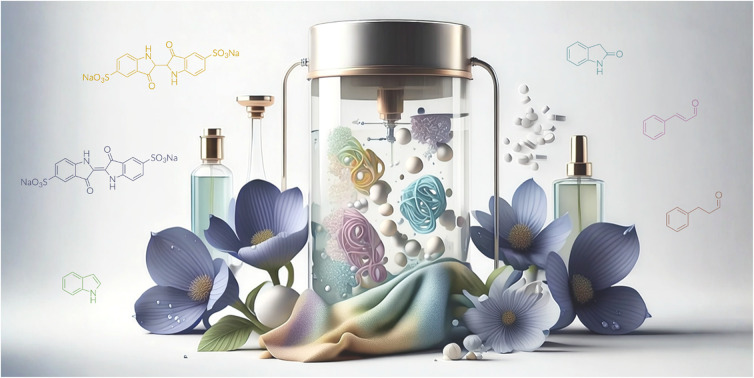

## Introduction

**Fig. 1:**
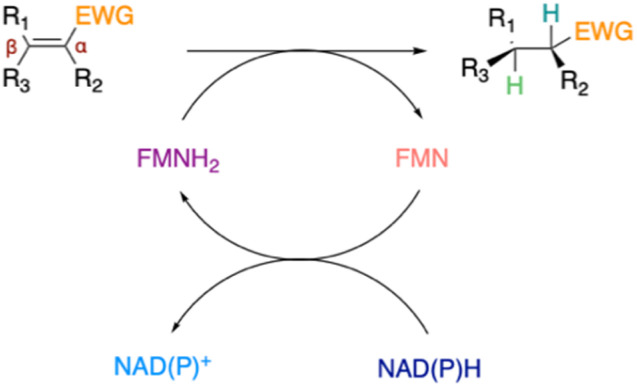
Ene-reductases FMN-dependent reduction of C=C bonds activated with EWG at the expense of NAD(P)H cofactor.^2^

The increasing demand for enantiopure molecules in the fine-chemical, pharmaceutical and fragrance industries triggered the development of well-characterised, efficient and stable catalysts for their production. One of the most widely used industrial reactions to access chiral compounds is the asymmetric reduction of alkenes.^1,2^ Approaches for saturation of carbon–carbon double bonds rely on asymmetric hydrogenation technology, or on organocatalysis.^1,2^ However, these approaches require special catalysis expertise and/or specialised equipment, expensive hydrogen source and/or high catalyst loadings, which dramatically increases process costs and reduce their industrial viability. Moreover, the metals used for catalytic hydrogenation are becoming less readily available due to their high geological rarity, high demand, and extraction challenges, which has stimulated the search for more sustainable ways to reduce carbon-carbon double bonds.^3^ Enzymatic approaches, especially ene-reductases (ERs)/old yellow enzymes (OYEs), have emerged as a viable method for regio- and chemoselective alkene reduction, leading to shorter synthetic routes towards not only high-added value but also bulk chemicals.^1–4^ These flavin-mononucleotide (FMN)-dependant enzymes can catalyse the stereoselective reduction of C=C bonds activated with electron-withdrawing groups (EWG) at the expense of a nicotinamide cofactor (Fig. 1).

In the past few years, ERs have become a hot research topic, with many innovative example showcasing their versatility.^4,5^ They can reduce not only α,β-unsaturated compounds activated in C_α_ by EWG such as carboxylic acids, esters, aldehydes, ketones, nitro or nitrile groups, but have also been shown to reduce oximes into imines, to perform reductive cyclisation of α,β-unsaturated compounds containing an additional electrophilic group to form chiral cyclopropanes,^4,5^ or even alkynes to alkenes followed by subsequent reduction to alkanes.^6^

In addition, they have been shown to work in the oxidative direction using O_2_ as an oxidant at 70 °C,^5^ or even be coupled to photoexcitation to form C–C bonds *via* stereoselective formal intramolecular hydroalkylation of non-activated alkenes.^7^ OYEs have also often been paired with dehydrogenases to form redox-neutral cascades without the requirement of an external electron donor,^1–4^ to transaminases,^4^ or to squalene hopene cyclases (SHC).^4,8^ Interestingly, alternative NAD(P)H regeneration systems relying on electrochemical reductions, photocatalysts, cheap hydride sources, cofactor analogues or even cofactor-free reductions with ERs have been reported.^1–4^ ERs have been used in several chemo-enzymatic cascades,^1,4,5^ including the preparative scale synthesis of the antiepileptic pregabalin with high enantiomeric excess (>99%) and good yields (69%),^9^ or the production of the powerful odorant decanal from 2*E*-decenal with 10 g L^−1^ of substrate.^10^ In addition, new OYE/ER homologues are becoming available through sequence similarity searches,^2,11^ site-directed mutagenesis or directed evolution towards desired substrates.^4,12,13^ However, despite the numerous applications of ERs at the lab scale, they are still underrepresented in industrial/preparative-scale reductions.^4^ While ERs have been immobilised in some studies with co-factor recycling enzymes partners (for example, glucose dehydrogenases (GDH)),^5,14–16^ applications of ERs in flow biocatalysis are even more scarce.^4–6^

Herein, to bring ERs closer to industrial applications requirements, we selected four promising ERs for rational immobilisation and explored the continuous biocatalytic transformation of cinnamaldehyde into 3-phenylpropionaldehyde. Then, to broaden the scope of potential applications of ERs, we propose a novel approach for sustainable dyeing at neutral pH with an enzymatic cascade to convert indole into leuco-indigo, the reduced water-soluble form of indigo that can intercalate into the fibres before being air oxidised back to the everlasting indigo.^17,18^

## Experimental section

### Reagents

All chemical reagents were acquired from ThermoFisher Scientific, Fluka (Honeywell), Sigma Aldrich (Merck) or VWR (Avantor) unless otherwise specified. Cofactors involved in biotransformations (NADH, NAD^+^, NADPH and NADP^+^) were acquired from Apollo Scientific. The HisTrapFF crude® column used for protein purification was purchased from Cytiva. All commercial chemicals were used without further purification. All enzymes were produced in house, except the *rAae*UPO peroxidase that was generously donated by the lab of Prof. Dr Frank Hollmann.

### Ene-reductases expression and purification

The pET28b(+) plasmids harboring the genes of the different ERs (synthetised by Microsynth) or of *Bm*GDH^19^ (Table S1 and Fig. S1) were transformed into chemically competent *E. coli* BL21 (DE3) cells for *Mc*OYE, *He*OYE, *Ph*ENR and *Bm*GDH or into *E. coli* BL21 Lemo21 (DE3) for *Tt*ENR. Then, 10 mL of LB media (yeast extract (5 g L^−1^), tryptone or N-Z amine (10 g L^−1^), NaCl (10 g L^−1^)) supplemented with the appropriate antibiotic(s) (Table S2) was inoculated with a single colony and incubated overnight at 37 °C and 150 rpm. 1 mL of this preculture were then added to 300 mL of LB or TB supplemented with the appropriate antibiotics and with 500 µM l-rhamnose for *Tt*ENR (Table S2) and cells were then grown at 37 °C and 150 rpm until an OD_600_ of approximately 0.6–0.8 was reached. After that, the appropriate concentration of isopropyl β-d-1-thiogalactopyranoside (IPTG) was added to induce protein expression, and the flasks were incubated overnight at 25–37 °C (Table S2). Cells were then harvested by centrifugation (4500 rpm, 20 minutes, 4 °C), resuspended in 10 mL of loading buffer (Table S3) on ice and lysed by sonication at 40% amplitude for 8 min, with pulses of 5 s ON, 10 s OFF. After centrifugation (12 000 g, 4 °C, 45 min), the supernatant was filtered with a 0.45 µm filter, and the different enzymes were then purified by metal affinity chromatography (IMAC) using an AKTA™ Start. Pure fractions were pooled and dialysed for 20 h at 4 °C with dialysis buffer (Table S3) with one buffer exchange after 2 h. Purification of the enzyme was checked by analysing the different fractions with a 12% SDS PAGE (Fig. S2) and concentration of the purified enzymes was estimated measuring the absorbance at 280 nm in the EPOCH2 (nanodrop Take3plate) using extinction coefficients (*ε*) and molecular weights of predicted using the ExPASy ProtParam tool (Table S1).^20^ Low protein concentrations (<1 mg mL^−1^) were determined by Bradford assay by measuring the absorbance at 595 nm of a solution containing 5 µL of protein sample with 250 µL of Bradford reagent.

### Activity assays

The activity of ERs was determined by monitoring the decrease in absorbance of NAD(P)H at 340 nm (*ε* = 6220 mol^−1^ L cm^−1^) in a 1 mL cuvette at 25, 30 or 75 °C for 10 minutes. A typical reaction mixture contained 100 µL of NADPH (2 mM), 50 µL of maleimide (400 mM), 800 µL of phosphate buffer (100 mM, pH 7.4) containing 20% DMSO and 50 µL of enzyme with appropriate dilution. Cofactor was incubated for 10 minutes with the enzyme at optimal assay's temperature before the assay was triggered by the addition of substrate. During the investigation of the optimal pH for activity assays, citrate buffer (100 mM, pH 5.4), phosphate buffer (100 mM, pH 7.4), or bicarbonate buffer (100 mM, pH 9.4) containing 20% of DMSO were used.

The activity of *Bm*GDH^19^ was determined by monitoring the increase in NADPH absorbance at 340 nm (*ε* = 6220 mol^−1^ L cm^−1^) in a 1 mL cuvette at 37 °C for 10 minutes. A typical reaction mixture contained 100 µL of NADP^+^ (2 mM), 50 µL of enzyme with appropriate dilution and 850 µL of glucose (40 mM) in phosphate buffer (100 mM, pH 7.4). Cofactor was incubated for 10 minutes at 37 °C with the enzyme before the assay was triggered by the addition of glucose.

The activity of the *rAae*UPO peroxidase was determined by monitoring the increase in absorbance of ABTS (2,2′-azinobis(3-ethylbenzothiazoline-6-sulfonic acid)) at 420 nm (*ε* = 36 000 mol^−1^ L cm^−1^) in a 96-well plate at 25 °C for 10 minutes. A typical reaction mixture contained 20 µL of ABTS (5 mM), 20 µL of H_2_O_2_ (2 mM), 155 µL of sodium citrate buffer (100 mM, pH 4.4), and 5 µL of the enzyme at the appropriate dilution (0.01 mg mL^−1^). The assay was triggered by the addition of hydrogen peroxide.

All activity tests were performed in triplicates and one unit [U] is defined as the amount of enzyme which catalyses the formation or depletion of 1 µmol of product or substrate per minute. To determine enzymatic activity, 20 mg of immobilized enzyme was added to 10 mL of a substrate mixture (containing substrate and cofactor concentrations identical to the free enzyme assays). The progress of the reaction was monitored by sampling the mixture at 2-minute intervals and measuring the absorbance of NAD(P)H at 340 nm.

### Enzyme immobilisation

Enzymes were immobilised using two primary strategies: directed immobilisation, through heterogenous supports modified with metal-chelates and epoxy functionalities and covalent attachment, using either epoxy or aldehyde modified resins. The cobalt-chelated and aldehyde-functionalised supports were prepared as previously described.^21^

For immobilisation *via* metal affinity and epoxy chemistry, the respective metal-chelated resin was suspended in an enzyme solution prepared in potassium phosphate buffer (50 mM, pH 8.0) to reach the desired loading of enzyme to support in a 1 : 10 resin-to-solution ratio (w/v). All suspensions were incubated for 16 hours at RT with orbital shaking. Following incubation, the immobilised preparations were washed with 3 volumes of desorption buffer (EDTA (50 mM), NaCl (500 mM) in phosphate buffer (50 mM, pH 7.2)) to remove the metal and incubated overnight with a 3 M glycine solution (3 M) to block any unreacted epoxy groups. For the aldehyde immobilisation, the solution of protein was added to the support and incubated at room temperature for at least 4 h. After the incubation, 5 volumes of NaBH_4_ (1 mg mL^−1^) were used to reduce the formed imines, and the resin subsequently washed with abundant water.

*Bm*GDH was immobilised following a previously reported protocol.^19^

All immobilised biocatalysts were washed thoroughly with their respective buffers after immobilisation and stored at 4 °C until used.

The immobilisation yield (IY) was determined by measuring the protein concentration *via* the Bradford assay before and after the immobilisation procedure. The initial protein concentration (*E*_i_) was measured from the enzyme solution prior to adding the support. After immobilisation, the supernatant containing the unbound enzyme was collected, and its protein concentration was measured (*E*_f_). The immobilisation yield was expressed as a percentage using the following formula:IY (%) = 100 × (*E*_i_ − *E*_f_)/*E*_i_

Recovered activity (RA) measures the percentage of the enzyme's original catalytic activity that is retained after it has been immobilised onto the support.

The recovered activity is then calculated as a percentage using the following formula:RA (%) = [*A*_immo_/(*A*_sol_ × loading × IY)] × 100where: *A*_immo_ = measured activity of the immobilised enzyme (U per g support), *A*_sol_ = specific activity of the free enzyme (U per mg enzyme). Loading = initial enzyme offered for immobilisation (mg enzyme per g support).

### Batch biotransformations

Initial screenings of ER-mediated reduction of cinnamaldehyde in batch were performed for 24 hours under shaking at 25 °C in phosphate buffer (100 mM, pH 7.4) containing 20% of DMSO in a total reaction volume of 1 mL, in the presence of cinnamaldehyde (1 mM), NAD(P)H (2 mM) without cofactor recycling system. 0.1 mg mL^−1^ of the free enzymes was used, and equivalent amounts of enzyme units was used for both soluble and heterogenous biotransformations. At the 10 mM scale, NAD(P)H (1 mM) was used with *Bm*GDH as recycling enzyme partner and glucose (40 mM) as sacrificial substrate using the same conditions.

Biotransformations aiming to convert indole into indigo were performed for 2–24 hours under shaking at 25 °C in citrate buffer (100 mM, pH 4.4) or phosphate buffer (100 mM, pH 7.4) containing 10% of acetonitrile in a total reaction volume of 1 mL, in the presence of indole (1–50 mM), 1–5 equivalents of H_2_O_2_ or cumene hydroperoxide, and 0.05 mg mL^−1^ of *rAae*UPO (3 U). Controls were run in the presence of all reaction components, except the enzyme. Indigo dyeing was performed by prewetting the tissue with water, soaking it into the reducing bath for 2–24 hours at 25 °C under shaking, removing it and letting it air oxidise, washing it and finally neutralising by soaking it with acetic acid. For chemical reductions, the oxidating bath was composed of sodium dithionite (Na_2_S_2_O_4,_ 40 mM) or glucose as reducing agents at pH 12 (NaOH, 200 mM), whereas enzymatic reductions were performed at pH 7.4 using 1 mg mL^−1^ of each ER, 0.5 mg mL^−1^ of glucose dehydrogenase from *Bacillus megaterium* (*Bm*GDH) and glucose (40 mM) to recycle 1 mM of NAD(P)H, keep the system under reduced conditions and potentially outcompete air oxidation. A layer of mineral oil was also added on the top of each reaction before each solution was bubbled with nitrogen to help in this purpose. Both chemical and enzymatic reductions were performed using 0.5 or 1 mM of indigo or indigo carmine.

### Flow biotransformations

For flow biotransformations, 1.5 g of immobilised *He*OYE was mixed with 1.4 g of *Bm*GDH (activity ratio 2 : 1) in a packed-bed reactor, and flow reactions were performed at 30 °C in phosphate buffer (100 mM, pH 7.4) containing 20% of DMSO with 30 minutes residence time (0.1 mg mL^−1^ flow rate in a 3 mL mixed bed reactor), in the presence of cinnamaldehyde (10 mM), NAD(P)H (1 mM) and glucose (40 mM). Space-time yield (STY, [g L^−1^ h^−1^]) was calculated by dividing the amounts of grams produced in batch or in flow by the time required to reach steady-state conversion. Reactions were performed at each enzyme's optimal working temperature: 30 °C for *Mc*OYE and *He*OYE, and 75 °C for *Tt*ENR.

### Analytics

HPLC samples were prepared by adding 50 µL of the reaction mixture to 225 µL of HCl (0.1%) and 225 µL of acetronitrile. The samples were filtered (0.45 µm) and analysed by HPLC (Dionex UltiMate 3000, Waters X-Bridge C18 (3.5 µm, 2.1 Å ∼100 mm)), eluent: A (water + 0.1% trifluoroacetic acid) and B (MeCN + 0.1% trifluoroacetic acid). The sample was run at 0.8 mL min^−1^ at 45 °C with a gradient from 5 : 95 *A* : *B* to 95 : 5 *B* : *A* over 4 minutes. While indole and 2-oxindole could be easily analysed by HPLC, indigo proved to be a very challenging compound to analyse because of its insolubility in almost all organic solvents. Its conversion was therefore determined by simply weighting the precipitate (10–50 mM scale) or dissolving it in DMSO followed by absorbance measurements at 620 nm (0.1–5 mM scales).

## Results and discussion

### Ene-reductases selection, expression and purification

Among the significant diversity of available ERs, we focused on those stemming from extremophilic organisms, or showing homology with thermophilic ERs identified based on three-dimensional constellations of functional groups in active sites by Steinkellner *et al.*,^11^ aptly termed “thermophilic like” OYEs or ERs.^2^ First, the isoform 1 of a thermophilic-like OYE from *Mucor circinelloides* (*Mc*OYE) was selected because of its ability to efficiently reduce classical OYE substrates in whole-cell biotransformations (Table S1, entry 1).^22^ Then, as previous examples of enzymes extracted from the halophile *Halomonas elongata* showed excellent expression profiles and good performance in our group,^23^ we identified an ER gene found in the genome of *H. elongata* that we named *He*OYE (Table S1, entry 2).^24^ Finally, we selected the two thermophilic ERs from *Pyrococcus horikoshii* (*Ph*ENR) and *Thermus thermophilus* (*Tt*ENR) with completely different fold but similar active sites as OYE because of their extremophilic character and inverted enantiopreference as compared to classical OYEs (Table S1, entries 3 and 4).^11^ While *Ph*ENR and *Tt*ENR were previously characterised, *Mc*OYE was never purified and *He*OYE never expressed nor purified. The four ERs were successfully expressed and purified (Fig. S1A and B). While *Mc*OYE, *He*OYE and *Ph*ENR could be expressed using *E. coli* BL21, *Tt*ENR had to be expressed in *E. coli* BL21 Lemo21 (Fig. S3). Because it was found in the insoluble fraction when *E. coli* BL21 was used as expression strain. While good to excellent expression yields were obtained for *Mc*OYE and *He*OYE, only moderate expression yields were obtained for *Ph*ENR and *Tt*ENR (Fig. S2C).

### Substrate scope

Optimal reactions conditions to establish standard activity assays were identified (Fig. S4). While *Mc*OYE and *Tt*ENR were found to be NADPH-dependent as in previous studies,^11,22^ with only traces of activity detected with NADH (<10%), *He*OYE strongly preferred NADH with no activity detected with NADPH. Surprisingly, even though a small activity was detected in the crude extract of *Ph*ENR, no activity was detected using the purified *Ph*ENR with either cofactors, nor with any of the tested substrates (Table 1). Activity of *Tt*ENR and *Ph*ENR was previously reported at pH of 7.^11^ Our investigations revealed maximum activity at pH 7.4 for both *Mc*OYE and *He*OYE, in agreement with the growth pHs of their respective organisms (Fig. S4).^23,24^ As expected, the optimal temperature for *Mc*OYE and *He*OYE stemming from mesophilic/halophilic organisms was found to be 30 °C, while the thermophilic *Tt*ENR exhibited maximum activity at 75 °C. With the purified ERs in our hands, we investigated their respective substrate scope using optimised conditions. While several substrate concentrations were tested, only the ones resulting in the best activity are highlighted in Table 1. Not surprisingly, substrates activated with the most EWGs such as *trans*-β-nitrotyrosol (Table 1, entry 10) and maleimide (Table 1 entry 7) resulted in higher activity, and *He*OYE tends to have higher activity than *Mc*OYE, followed by *Tt*ENR which is generally less active. Surprisingly, *He*OYE showed no activity with *trans*-β-nitrotyrosol, while only minimal activities were detected with 4-phenylbut-3-yn-2-one.

**Table 1:** Substrate scope and respective activities of the ERs investigated in this study. Activity assays were conducted in the presence of NAD(P)H (0.2 mM), 1–20 mM of the respective substrates, in phosphate buffer (100 mM, pH 7.4) containing 20% of DMSO. *Mc*OYE and *Tt*ENR were found to be NADPH-dependent, while *He*OYE preferred NADH as cofactor. No activity was ever detected with *Ph*ENR. Activity assays were conducted at 75 °C for *Tt*ENR, and at 30 °C for *Mc*OYE and *He*OYE

Entry	Substrate	Structure	*C*_substrate_ [mM]	*A*_*Mc*OYE_ [U per mg]	*A*_*He*OYE_ [U per mg]	*A*_*Tt*ENR_ [U per mg]	*A*_*Ph*ENR_ [U per mg]
1	2-Cyclohexenone	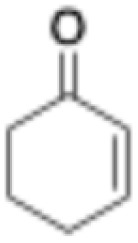	20	0.4 ± 0.1	2.1 ± 0.4	0.6 ± 0.1	0
2	2-Methyl-cyclohexenone	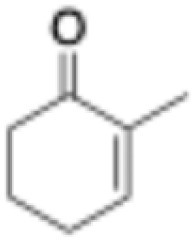	20	0.6 ± 0.1	1.0 ± 0.1	0.8 ± 0.2	0
3	2-Cyclopentenone	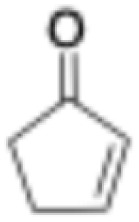	20	0.3 ± 0.1	1.7 ± 0.2	0.7 ± 0.1	0
4	4-Keto-isophorone	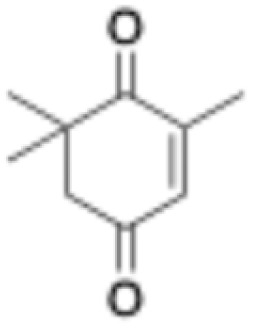	10	1.4 ± 0.2	2.7 ± 0.3	0.7 ± 0.2	0
5	Citral	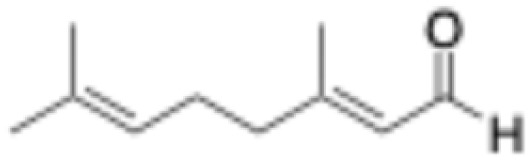	1	0.4 ± 0.1	0.7 ± 0.1	0.3 ± 0.1	0
6	Cinnamaldehyde	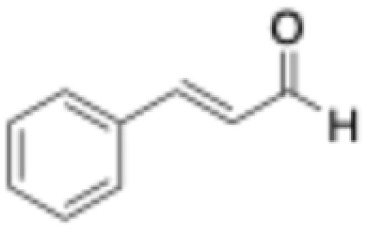	1	3.5 ± 0.4	3.1 ± 0.1	1.1 ± 0.2	0
7	Maleimide	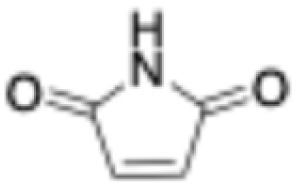	20	8.8 ± 0.6	37.1 ± 0.9	4.5 ± 0.4	0
8	(*R*)-(−)-Carvone	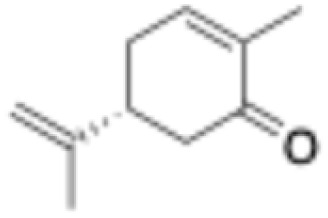	10	2.3 ± 0.3	0.4 ± 0.1	0.3 ± 0.1	0
9	(*S*)-(+)-Carvone	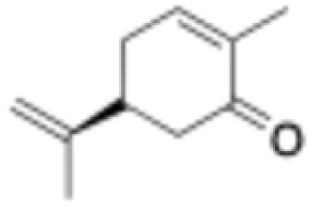	10	1.1 ± 0.2	0.4 ± 0.1	0.5 ± 0.1	0
10	*trans*-β-Nitrotyrosol	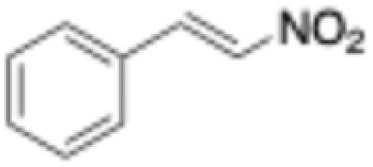	1	18.5 ± 0.9	0	5.9 ± 0.8	0
11	Ciannamonitrile	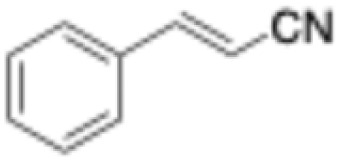	1	0.4 ± 0.1	0.6 ± 0.2	0.2 ± 0.1	0
12	4-Phenylbut-3-yn-2-one	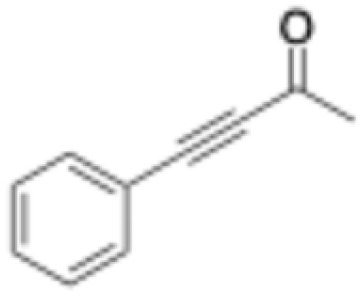	1	0.1 ± 0.1	0.1 ± 0.1	0.1 ± 0.1	0

### Heterogenous biocatalyst preparation

To design an efficient immobilized system, we analysed the surface properties of *Mc*OYE, *Tt*ENR, and *He*OYE using CapiPy^25^ to guide the choice of immobilisation chemistry. We focused on the lysine coverage (percentage of the surface occupied by lysine residues) as well as the position of the His-tag, which is in all cases located at the N-terminal (Fig. S5). *Mc*OYE (9% lysine coverage, Fig. S5A) contains two peripheral lysine clusters, as well as three negative clusters (Fig. S5). *Tt*ENR (7% lysine coverage) has lysines distributed across the surface, with the His-tag positioned far from both active sites in this dimeric enzyme (Fig. S5B). *He*OYE has only four surface lysines (1%), one near the active site, limiting covalent attachment options and risking catalytic site obstruction and the His-tag is located at the N-terminus, and is positioned close the active site (Fig. S5B).

Given the presence of the His-tag and favourable positioning of it, especially in *Mc*OYE and *Tt*ENR, all enzymes were immobilised on epoxy-metal support. This enables a first rapid His-tag coordination to cobalt followed by a slower covalent bond of the surface lysines in the proximity to the epoxy moiety of the support. Table 2 presents the results of the selected optimal immobilisation for each enzyme. *Tt*ENR and *Mc*OYE achieved >99% immobilization yield at 1 mg g^−1^ loading. *Tt*ENR showed full activity retention with EP400/SS, likely due to pre-orientation that left both active sites accessible (immobilisation screening in Table S5). *Mc*OYE showed 75% retained activity with HFA403/S, a long arm amino-epoxy methacrylate resin, which will position the protein guided by the His-tag as well as the previously mentioned negative clusters specially those located down from the active site and in close proximity to the N-terminal (Fig. S6b, immobilisation screening Table S4).

**Table 2:** Selected immobilisation results for *Tt*ENR, *Mc*OYE and *He*OYE. All enzymes were immobilised on heterogeneous supports with epoxy-metal binding chemistry. IY: Immobilization Yield, RA: Recovered Activity

Enzyme	Support	Loading (mg g^−1^)	IY	RA	U per g
*Tt*ENR	EP400/SS	1	>99%	100%	2.6 ± 0.1
*Mc*OYE	HFA403/S	1	>99%	75%	6.6 ± 0.2
*He*OYE	6BCL	1	60%	23%	5.5 ± 0.3

For the immobilisation of *He*OYE, on the other hand, both aldehyde and epoxy-metal supports were tested (Table S6). Low immobilisation yields were obtained with high loading (5 mg g^−1^), possibly due to the presence of only 3 exposed lysines. Lowering the loading to 1 mg g^−1^, allowed for yields over 60% with all different resins, but the immobilised enzyme was less active, achieving our maximum with agarose support and a 23% recovered activity. Noteworthy, silica materials showed a very good immobilisation yield and recovered activity, but the reusability of the catalyst was compromised because of abrasion of the material (Table S6, entries 8 and 17).

These results confirm that dual-mode affinity-covalent immobilisation efficiency depends strongly on surface architecture, with optimal His-tag placement and distal lysines being key to high yield and activity preservation.

### Flow biocatalytic synthesis of 3-propionaldehyde

To test the efficiency of ER-mediated biocatalysis in flow, we chose the readily available and easy to monitor cinnamaldehyde, whose reduced product 3-phenylpropionaldehyde (or hydrocinnamal-dehyde) is used in perfumes, as flavouring agent, as building block in organic synthesis, in agrochemicals or for pharmaceuticals,^26^ and has even shown interesting antibacterial properties.^27^ Initial screenings of batch reactions using the free and immobilised enzymes at a 1 mM scale, using NAD(P)H (2 mM) without cofactor recycling system revealed that excellent conversions were reached in all cases (89–100%) although with longer time for *Tt*ENR and for the immobilised *Mc*OYE (Table 3 entries 1–6 and Fig. S7).

**Table 3:** ER-mediated reductions of cinnamaldehyde in phosphate buffer (100 mM, pH 7.4) containing 20% of DMSO with or without NAD(P)H recycling system in the presence of free/immobilised *Tt*ENR, *Mc*OYE or *He*OYE. Reactions without recycling system were performed at the 1 mM scale in the presence of NAD(P)H (2 mM) and 0.1 mg mL^−1^ of the free enzyme or equivalent amounts of immobilised enzyme units. Reactions with recycling system (A) were performed at the 10 mM scale in the presence of NAD(P)H (1 mM), 2 units of *He*OYE : 1 unit of *Bm*GHD ratio and glucose (40 mM) as sacrificial substrate. For flow biotransformations (B), a residence time of 30 minutes was used. Reactions were performed at each enzyme's optimal working temperature: 30 °C for *Mc*OYE and *He*OYE and 75 °C for *Tt*ENR. Conversions were determined by HPLC analysis based on product formation

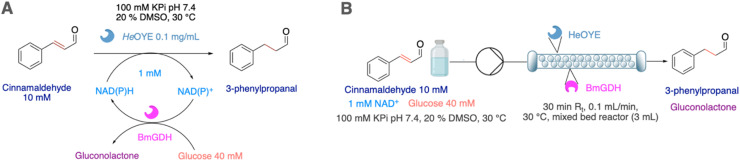							
Entry	Type	Enzyme	Scale [mM]	Recycling system	Rxn time [h]	Conversion [%]	Space-time-yield [mg L^−1^ h^−1^]
1	Batch	Free *Tt*ENR	1	—	18	100	7.5
2		Immobilised *Tt*ENR			24	100	5.6
3		Free *Mc*OYE			2	89	59.7
4		Immobilised *Mc*OYE			18	89	6.6
5		Free *He*OYE			2	98	65.8
6		Immobilised *He*OYE			2	97	65.1
7		Free *He*OYE	10	*Bm*GDH	24	62	34.7
8		Immobilised *He*OYE			24	62	34.7
9	Flow	Immobilised *He*OYE			0.5	81	2173.9

*He*OYE was further investigated in flow biocatalytic transformations in a mixed-bed reactor co-housing immobilised glucose dehydrogenase from *Bacillus megaterium* (*Bm*GDH) using a 2 units of *He*OYE : 1 unit of *Bm*GHD ratio, glucose (40 mM) and 0.1 equivalents of NAD(P)H. While batch biotransformations with the free and immobilised enzymes resulted in 60% conversion after 24 hours (Table 3 entries 7 and 8), 80% conversion was reached in flow and the biocatalyst was stable over 6 cycles with residence times of 30 minutes (Table 3 entry 8, Fig. S8). The space time yield (STY) for the production of 3-phenylpropionaldehyde could thus be increased from 34.7 [mg L^−1^ h^−1^] in batch to 2173.9 [mg L^−1^ h^−1^] in flow, which represents a 62-fold process intensification.

### Sustainable indigo dyeing

**Fig. 2:**
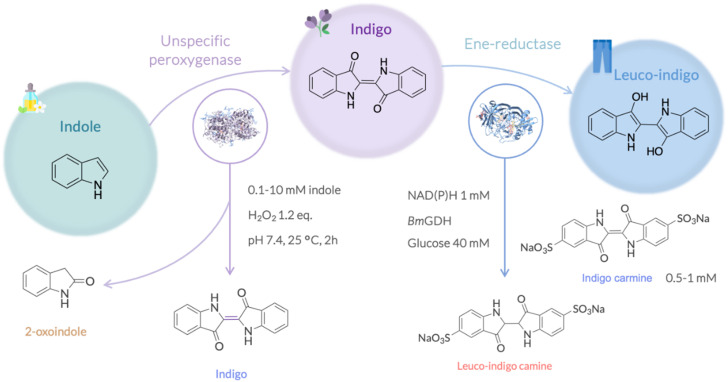
Enzymatic cascade for indigo synthesis and further reduction to leuco-indigo.

Among substrates containing an α,β-unsaturated motif activated in C_α_ by EWGs, indigo is certainly one of the most widely used worldwide for textile dyeing. To intercalate into the fibres, this insoluble pigment needs to be reduced to the water soluble alkaline leuco-indigo that will subsequently be air oxidised back to the everlasting indigo.^17,18^ Typical chemical reducing agents used in this process such as sodium dithionite result in environmentally hazardous sulfate by-products that can also corrode pipes. Promising microbial route using recombinant whole-cell biocatalysts^28,29^ or electrochemical methods reduction processes^30^ have emerged more recently, and biocatalysis in particular could offer a greener and less energy-intensive alternative.^31,32^ The combination of an unspecific peroxygenase with reductases is a promising route. In fact, compared to other enzymes such as P450 monooxygenases that can perform similar functions, UPOs do not require expensive electron donors and use H_2_O_2_ as co-substrate and oxidant agent, which make them particularly appealing for the synthesis of valuable industrial products (Fig. 2).^33^

### UPO-mediated synthesis of indigo and 2-oxindole from indole

Following the method of Ullrich *et al.*^33^ with used the unspecific fungal oxidase from *Agrocybe aegerita* (*rAe*UPO, PaDa-I variant) available in house. The reaction could be followed by TLC as well as visually, with the solution rapidly turning blue to amber-greenish with a blue precipitate appearing after 30 minutes (Fig. S9A). We performed the reaction at different scales, however, indigo conversions were low (<5%) (Table S7). Almost complete indole depletion was observed in all cases (Fig. S9C and D), and while the presence of indigo was confirmed with standard samples (Fig. S9B), the main product was identified to be 2-oxindole (64–85% conversion, Table S7). In an attempt to increase indigo's yield, we screened different H_2_O_2_ equivalents, pHs (4.4 and 7.4) as well as an alternative oxidant (Table S8), but none of the tested conditions resulted in any improvement of indigo conversion. Ullrich *et al.*^33^ reported that different UPOs showed quite different preference regarding the formation of 2-oxindole or indoxyl (3-oxindole), suggesting a direct influence of the UPOs active site on the 2-3-epoxide opening. However, 2-oxindole itself has been reported to be a valuable precursor for many pharmaceutically-relevant compounds such as nintedanib, ziprasidone or ropinirole.^34^

### ER-mediated reduction of indigo into leuco-indigo

Reduction of indigo into leuco-indigo was previously reported with azo-reductases (AZs)^31,35,36^ but never using ERs. ERs sequence alignments with these enzymes resulted in low dope scores (0.006–0.03), <15% identity, and different residues composing their respective active sites, thereby indicating that AZRs and ERs are fundamentally different enzymes. While no activity was detected in the control in the absence of indigo and indigo carmine, low (0.1–0.3 U per mg) and moderate (0.7–2.5 U per mg) activities were detected using *Mc*OYE, *He*OYE and *Tt*ENR with indigo and indigo-carmine, respectively (Table S9). Even though these activities are lower than the ones reported using AZRs (30–66 U per mg), the latter mainly work at basic pH whereas the reduction with ERs is performed at neutral pH.

**Fig. 3:**
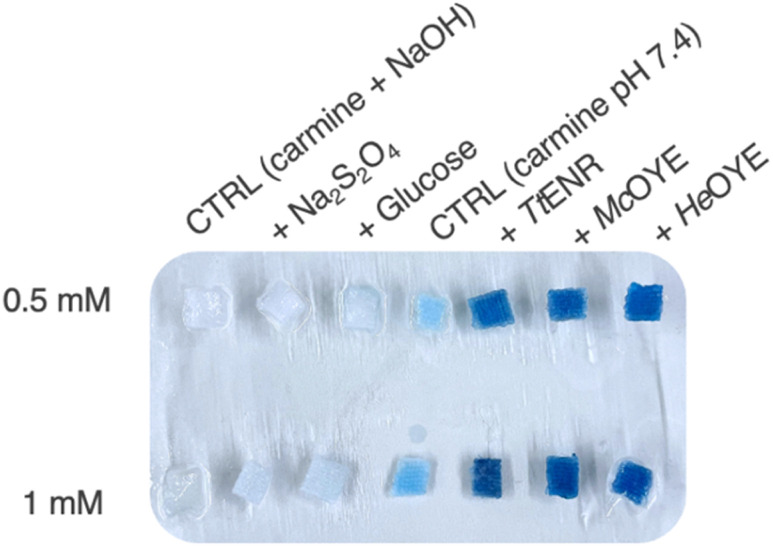
Chemical and enzymatic dyeing of pieces of cotton dyeing with 0.5 and 1 mM indigo carmine after washing and neutralisation. Chemical dyeing was performed at pH 12 in the presence of Na_2_S_2_O_4_ (40 mM) or glucose (40 mM), NaOH (200 mM), and enzymatic reactions were performed in the presence of 1 mg mL^−1^ of ene-reductase, 0.5 mg mL^−1^ of glucose dehydrogenase, glucose (40 mM) and NAD(P)H (1 mM).

To compare the efficacy of ERs with conventional indigo dyeing processes, in the absence of better analytical methods, we dyed cotton cloth chemically at pH 12 using sodium dithionite (Na_2_S_2_O_4_, 40 mM) or glucose (40 mM) as reducing agents, since both were previously reported to reduce indigo.^37^ Using indigo, only the chemical reduction at basic pH resulted in dyeing (Fig. S10A and Table S10 entries 1–6), most probably because it could not be solubilised enough to reach the active site of ERs. When the more soluble indigo carmine was used instead, reduction into the soluble yellow form was observed in all cases after 2 hours (Fig. S10B), but in the case of chemical dyeing, this colouration was show to be due to dye degradation (Table S10 entries 7–9), whereas the system stayed yellow with the ER-mediated reactions, even after 1 week (Fig. S10C and Table S10 entries 10–12). The three enzymes (*Tt*ENR, *Mc*OYE and *He*OYE) were indeed able to efficiently dye the cotton pieces at neutral pH, while chemical methods failed (Fig. 3). While indigo dyeing itself would require optimisation of the solubility conditions using for instance a biphasic system, this process could be very promising for textile dyeing using indigo derivatives with many colour shades that could be synthetised using indole derivatives (Fig. S11).^33^

## Conclusions

In conclusion, in this work, we expanded the repertoire of enzymes available for the synthesis of chiral molecules by successfully expressing and purifying a novel ene-reductase from the halophilic organism *Halomonas elonga*. Besides being expressed with excellent yields and exhibiting moderate activity towards almost all of the substrates, *He*OYE was shown to perform well in flow, with a 62-fold process intensification compared to similar batch synthesis of 2-phenylpropionaldehyde from cinnamaldehyde and an increase of conversion from 62 to 81%, with the biocatalyst being stable over 3 hours. These first step into ER-mediated flow biocatalysis could pave the way to further studies at higher scale or to their integration in flow biocatalytic cascades. Moreover, while attempts of reducing indigo-carmine into leuco-indigo carmine using chemical methods resulted in dye degradation, three of the four extremophilic/extremophilic-like ERs proved to efficiently perform that reaction at neutral pH. This is the first example of the ER-mediated dyeing. By being able to produce small quantities of indigo from indole with *rAae*UPO and co-producing the valuable 2-oxindole precursor of many pharmaceutically relevant molecules, we provide a proof-of-concept of a sustainable enzymatic cascade's feasibility for indigo derivates dyeing at neutral pH. Many possibilities remain open for further enzyme engineering, optimisation/scale-up of the process or ways to assemble the cascade, such as the *co*-immobilisation of the three enzymes (UPOs, ERs and GDH) for an *in situ* one pot dyeing process in batch, or even in flow. Our strategy could even be applied to other dyes with different colors starting from functionalised indole. Overall, both flow biocatalytic and dyeing approaches using *He*OYE are innovative, safe, green and versatile strategies for the reduction of carbon–carbon double bonds, which could pave the way to the effective implementation of biocatalysis at the industrial scale, while alleviating the ecological imbalance of conventional (electro)chemical reductions.

## Author contributions

F. P. supervised and guided the project, M. B. performed initial selection of ERs, InSEIT rationally immobilised all enzymes, L. P. conceptualised the idea, performed most of the experimental work and wrote the initial draft. All authors discussed and agreed to the final version of the manuscript.

## Conflicts of interest

There are no conflicts to declare.

## Supplementary Material

RA-015-D5RA06869J-s001

## Data Availability

The data supporting this article have been included as part of the supplementary information (SI). Supplementary information is available. See DOI: https://doi.org/10.1039/d5ra06869j.
