# Supplementary material for: A novel ene-reductase from Halomonas elongata for flow biocatalytic synthesis of 3-phenylpropionaldehyde and sustainable indigo-carmine dyeing
Source: RSC Adv. 2025 Nov 12;15(51):43974–82. doi: 10.1039/d5ra06869j (PMC12610393; doi:10.1039/d5ra06869j)
Supplement: RA-015-D5RA06869J-s001 [file RA-015-D5RA06869J-s001.pdf]

Supplementary Information (SI) for RSC Advances.
This journal is © The Royal Society of Chemistry 2025

Supplementary Information

A novel ene-reductase from *Halomonas elongata* for flow biocatalysis of 3-phenylpropionaldehyde and sustainable indigo-carmine dyeing

Lauriane Pillet,[a] Cristina Lía Fernández Regueiro [b],Markus Richard Busch,[a] David Roura Padrosa,[b] and Francesca Paradisi*[a]

Table of contents

1. Enzymes selection, expression and purification 3

1.1 Enzymes selection 3

Table S1: Selected ene-reductases and their respective molecular weights, molar extinction coefficients and quaternary structures 3

1.2 Protein sequences and plasmid maps 3

Figure S1: Plasmid map of the *He*OYE gene in pET28b(+) backbone 4

1.3 Optimal expression and purification conditions 4

Table S2: Optimal expression conditions for each protein used in this study 5

Table S3: Optimised purification conditions for each protein used in this study 5

Figure S2: Representative SDS-PAGE gel of the purification fractions, of the purified ERs and expression yields 6

1.4 Optimisation of L-Rhamnose concentration for expression of *Tt*ENR in *E. coli* BL21 Lemo21 cells 6

Figure S3: SDS-PAGE gel of the optimisation of L-Rhamnose concentration for expression of *Tt*ENR in *E. coli* BL21 Lemo21 cells 7

2. Substrates scope 7

2.1 Optimisation of the activity assays conditions 7

Figure S4: Investigation of the optimal conditions for the activity of the ene-reductases 8

3. Bioinformatics and immobilised enzyme development 9

3.1 Bioinformatics analysis 9

Figure S5 Lysine surface coverage and N-terminal for the ene-reductases used in this study 9

Figure S6 Cluster analysis of *Mc*OYE 10

3.2 Immobilisation screening 10

Table S4 Immobilisation screening of *Mc*OYE 10

Table S5 Immobilisation screening of *Tt*ENR 11

Table S6 Immobilisation screening of *He*OYE 11

4. Flow biocatalytic synthesis of 3-phenylpropionaldehyde 12

4.1 Initial screenings with free and immobilised enzymes at the 1 mM scale 12

Figure S7: ER-mediated reductions of cinnamaldehyde in batch at the 1 mM scale without cofactor recycling system 12

4.2 Batch and flow biotransformations at the 10 mM scale with cofactor recycling system 13

Figure S8: *He*OYE-mediated reductions of cinnamaldehyde in batch and in flow at the 10 mM scale with cofactor recycling system 13

5. Sustainable indigo dyeing 13

5.1 UPO-mediated synthesis of indigo and 2-oxindole from indole 13

Optimisation of the H_2O_2 equivalents 13

Figure S9: Biotransformations of indole into indigo at the 1 and 5 mM scale using different equivalents of H_2O_2 after 2 hours 15

Table S7: Synthesis of indigo and 2-oxindole at different scales using different equivalents of H_2O_2 after 2 hours 16

Screening of another oxidant 16

Table S8: Synthesis of indigo and 2-oxindole from indole at different scales using cumene hydroperoxide as oxidant 17

5.2 ER-mediated reduction of indigo into leuco-indigo 17

Activity assay 17

Table S9: Specific activities of the different ERs towards indigo and indigo carmine 17
Comparison of chemical and ER-mediated dyeing 18
Figure S10: Comparison of chemical and enzymatic dyeing 18
Table S10: Chemical and enzymatic dyeing of 0.5 or 1 mM indigo or indigo carmine 19
Figure S11: Graphical abstract of the synthesis of indigo dyes from indole derivatives by using unspecific peroxygenases and their application for in-situ dyeing 19
6. Supplementary references 20

1. Enzymes selection, expression and purification

1.1. Enzymes selection

Table S1: Selected ene-reductases and their respective molecular weights, molar extinction coefficients and quaternary structures.[1-3] Extinction coefficients (ε) and molecular weights were predicted using the ExPASy ProtParam tool.[4] The quaternary structures were obtained from PDB files for *Ph*ENR, *Tt*ENR and *Bm*GDH, while for *Mc*OYE and *He*OYE they were predicted using Capipy.

Entry	Enzyme abbreviation	Full name and source organism	Mw [kDa]	ε [$mol^{-1}·L·cm^{-1}$]	Quaternary structure
1	*Mc*OYE	Old yellow enzyme from *Mucor circinelloides*	44	28420	Monomer
2	*He*OYE	Old yellow enzyme from *Halomonas elongata*	45	52830	Monomer
3	*Ph*ENR	Ene-reductase from *Pyrococcus horikoshii*	23	42985	Homodimer
4	*Tt*ENR	Ene-reductase from *Thermus thermophilus*	23	29910	Homodimer
5	*BmGDH*	Glucose dehydrogenase from *Bacillus megaterium*	29	29910	Homotetramer

1.2. Protein sequences and plasmid maps

Protein sequences of all enzymes:

*He*OYE:
MASMTGGQQMGRDPMTDTTLFTPFQLGSLSLPNRVIMAPLTRARTPDSVPGKLQQVYYEQRAGAGLIISEATNISPTARG
YVYTPGIWTDEQEAGWRQVVDAVHAKGGRIALQLWHVGRVSHEMVQPDGQQPVAPSALKGEGAQCFVEFEDGTAGR
HPTSTPRALETDEIPGIVEDYRQAAVRAKRAGFDMLEVHAANAYLLNQFLATGTNLRTDRYGGSIENRARFPLEVIDAVTEV
FGADRVGIRLTPFIEIFGLSDDEPEAMALYLAEQLDRRGLAYIHLNEPNWAGGDTTFPEGFRGRMREHFSGGLIYCGHYDAE
RAQQRLAENTADLIAFGRPFIANPDLPERLRTGAALNEPDQDTFYGGNEQGYTDYPFLDNGHDQTA

*Mc*OYE:
MASMTGGQQMGRDPMSSTKALFSPIKVGANTLKHRIVLAPLTRFRATPEAVPTDLQAKYYEQRASDGGLLVTEATFISRLA
GAYPQAPGIYNKEQIEGWKKATSAVHAKGGIFFLQLWHLGRVGSKYLNPNQEQVVSASDIPAPGKALTGADHEVPHALT
IDEIKSIVNDYAQAAKNAIEAGFDGVEIHGANGYLVDQFINSSSNNRTDIYGGSVENRGRFALEVVDAVVAAVGEERTAI
RFSPGNGFQGMFDDNVEETWGYLVSELQKNHPGLAYLHLIESRADLQTPDQKNTVDTLESYRKIWKGPFITAGGFSTSLE
FGNEIAEKTGDLVAYGRAFIANPDLPERLRNGWELNPYNRDTFYSHGAEGYTDYPFYNEKN

*Tt*ENR:
MRSYRAQGPLPGFYHYYPGVPAVVGVRVEERVNFCPAVWNTGLSADPPLFGVSISPKRFTHGLLLKARRFSASFHPFGQKD
LVHWLGSHSGREVDKGQAPHFLGHTGVPILEGAYAAYELELLEVHTFGDHDLFVGRVVAVWEEEGLLDEKGRPKPGLALLY
YGKGLYGRPAEETFAP

*Ph*ENR:
MGSDKIHHHHHHENLYFQGMEGYRLLYPMRTYLIVSGHGEETNVMAADWVTVVSFDPFIVGVAVAPKRTTHKLIKKYGEF
VISVPSLDVLRDVWIAGTKKGPSKLKEMSVTLIPSKKVKVPSIEEALANIECRVIDARSYGDHTFFVGEVVGYTYKDYAFEKGK
PNLKAKFLAHVSWSEFVTFSEKVHKAE

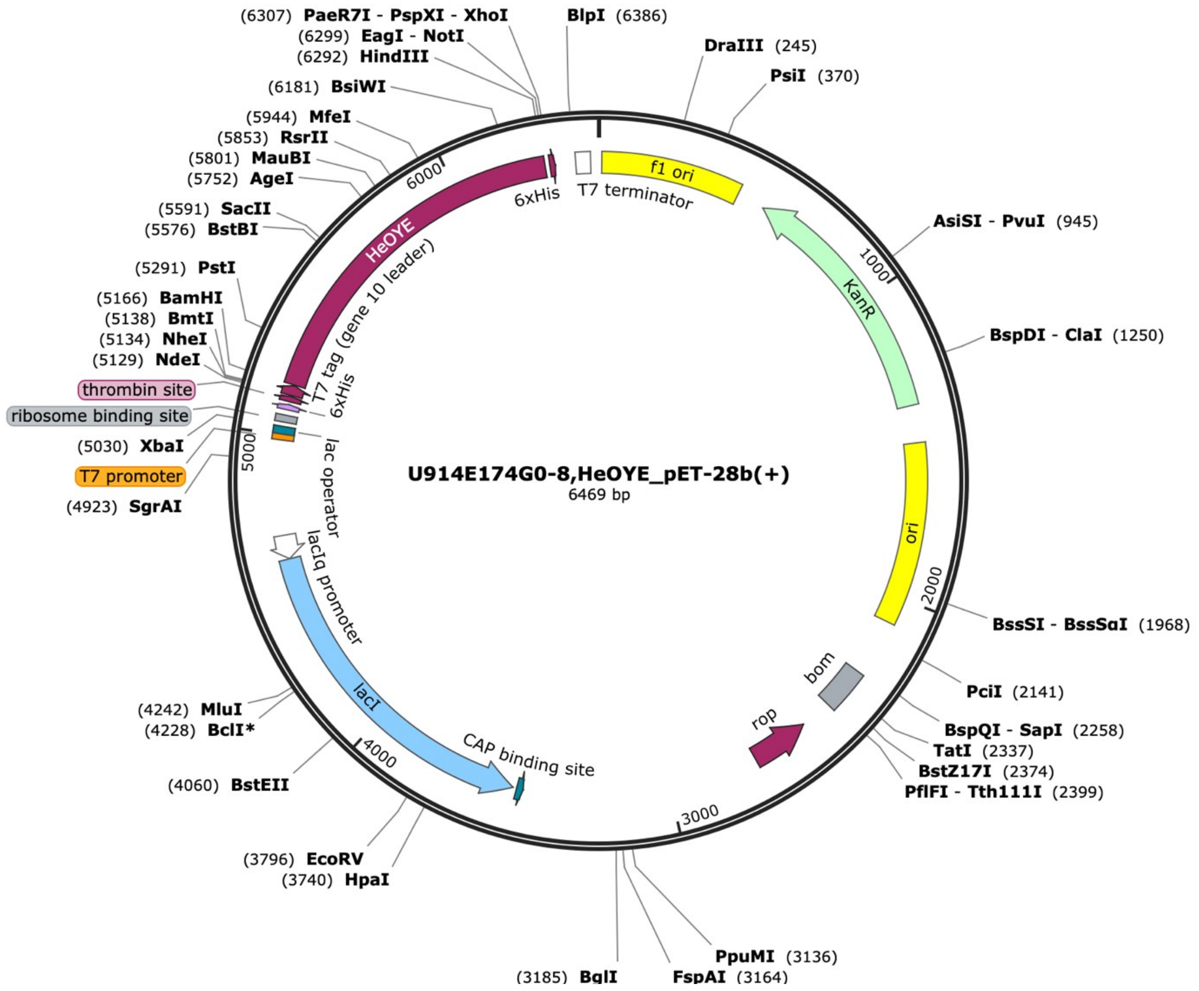

Figure S1: Plasmid map of the *He*OYE gene in the pET28b(+)backbone (plasmid synthetised by Microsynth).

1.3. Optimal expression and purification conditions

The four ERs and *Bm*GDH were expressed and purified as described in the experimental section, with the optimal expression conditions shown below in **Table S2**.

Table S2: Optimised expression conditions for each protein used in this study. Expression time was always overnight. Amp = ampicillin, Kan = kanamycin, Chlor = chloramphenicol. The working concentrations for ampicillin, kanamycin and chloramphenicol are 50, 100 and 25 μg/mL, respectively.

Enzyme	Plasmid backbone	*E. coli* cell line	Expression medium	Temperature after induction	Antibiotic
*Mc*OYE	pET28b(+)-	*E. coli* BL21 Star (DE3)	LB + 1 mM IPTG	25 °C	Kan
*He*OYE	pET28b(+)-	*E. coli* BL21 Star (DE3)	LB + 1 mM IPTG	25 °C	Kan
*Tt*ENR	pET28b(+)-	*E. coli* BL21 Star (DE3)	LB + 1 mM IPTG	25 °C	Kan
*Ph*ENR	pET28b(+)-	*E. coli* BL21 Lemo 21 (DE3)	LB + 0.5 mM L-rhamnose +0.4 mM IPTG	25 °C	Kan
*Bm*GDH	pET22b(+)-	*E. coli* BL21 (DE3)	TB + 1 mM IPTG	37 °C	Amp

Optimal purification conditions are shown in **Table S3**.

Table S3: Optimised purification conditions for each protein used in this study.

Enzyme	Column	*Loading buffer*	Elution buffer	Dialysis/storage buffer
*Bm*GDH	5 mL	100 mM phosphate buffer, 100 mM NaCl, 10 mM imidazole, pH 7.5	100 mM phosphate buffer, 100 mM NaCl, 300 mM imidazole, pH 7.5	100 mM phosphate buffer pH 7.5
*Mc*OYE and *He*OYE	5 mL	50 mM phosphate buffer, 100 mM NaCl, 30 mM imidazole, pH 7.4	50 mM phosphate buffer, 100 mM NaCl, 300 mM imidazole, pH 7.4	50 mM phosphate buffer pH 7.4
*Ph*ENR and *Tt*ENR	1 mL	50 mM phosphate buffer, 100 mM NaCl, 30 mM imidazole, pH 7.4	50 mM phosphate buffer, 100 mM NaCl, 300 mM imidazole, pH 7.4	50 mM phosphate buffer pH 7.4

All enzymes were successfully expressed and purified, with **Figure S2A** showing a representative SDS-PAGE analysis of the purification fractions (*He*OYE). While good to excellent expression yields were obtained for *Mc*OYE and *He*OYE, more moderate expression yields were obtained for *Ph*ENR and *Tt*ENR (**Figure S2C**).

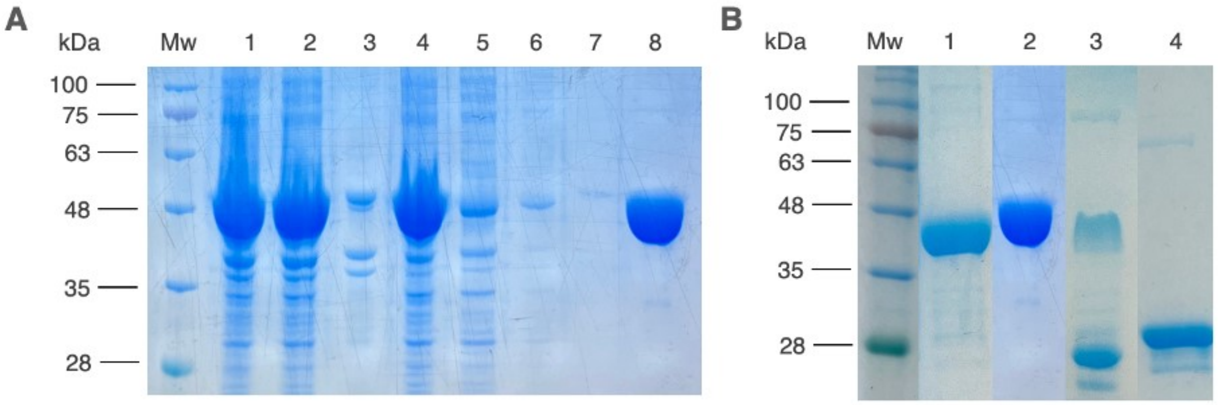

C

Enzyme	*E.coli* cell line	Expression yield [mg/$L_{culture}$]
*Mc*OYE	BL21 Star (DE3)	112
*He*OYE	BL21 Star (DE3	142
*Ph*ENR	BL21 Star (DE3	29
*Tt*ENR	BL21 Lemo21 (DE3	33

Figure S2: SDS-PAGE (12 % acrylamide) analysis after the expression in LB and purification of (**A**) *He*OYE in BL21 Star (DE3) at 25° overnight. Line 1: total protein before sonication. Line 2: total protein after sonication. Line 3: soluble protein fraction (crude extract). Line 4: insoluble protein fraction (pellet). Line 5: flowthrough after sample loading in the AKTA. Line 6: wash (with loading buffer). Line 7: wash unbound (with 10 % elution buffer). Line 8: purified protein and (**B**) Line 1: *Mc*OYE in BL21 Star (DE3) using 1 mM IPTG. Line 2: *He*OYE in BL21 Star (DE3) using 1 mM IPTG. Line 3: *Ph*ENR in BL21 Star (DE3) using 1 mM ITPG. Line 4: *Tt*ENR in BL21 Lemo21 (DE3) using 500 µM L-rhamnose and 0.4 mM IPTG. (**C**) Expression yields for the four purified ERs.

1.4. Optimisation of L-Rhamnose concentration for expression of *Tt*ENR in *E. coli* BL21 Lemo21 cells

While *Mc*OYE, *He*OYE and *Ph*ENR were expressed using *E.coli* BL21 Star in standard LB medium (**Figure S2**), *Tt*ENR was expressed in *E.coli* BL21 Lemo21 (**Figure S2**). *Tt*ENR was indeed found in the insoluble fraction when *E.coli* BL21 Star was used as an expression strain, and varying the levels of IPTG from 0.1 to 1 mM with the aim to obtain more properly folded proteins did not result in any improvement. While with no L-rhamnose, *Tt*ENR is almost exclusively found in the insoluble fraction (**Figure S3**, lines 1-2), L-rhamnose (500 µM) yielded enough soluble protein, without decreasing too much the total expression levels (**Figure S3**, lines 5-6).

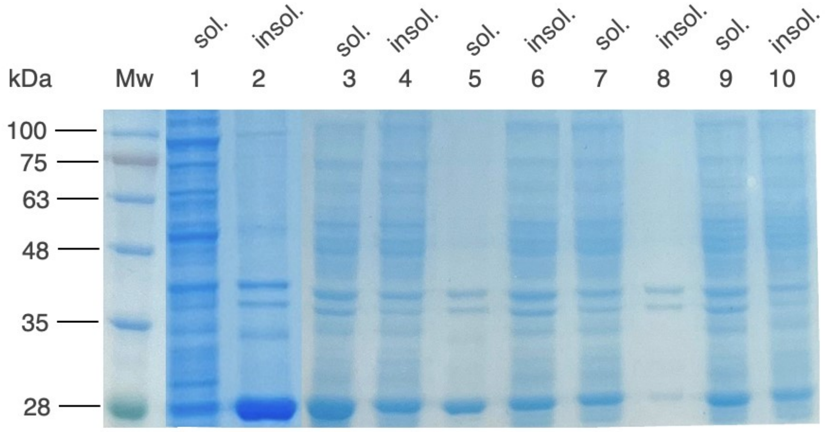

Figure S3: SDS-PAGE (12 % acrylamide) analysis of the soluble and insoluble fractions after the expression of *Tt*ENR in BL21 Lemo21 (DE3) at 25 °C overnight in LB medium using different concentrations of L-rhamnose and IPTG (0.4 mM) for induction. Lines 1 and 2: L-rhamnose (0 µM). Lines 3 and 4: L-rhamnose (250 µM). Lines 5 and 6: L-rhamnose (500 µM). Lined 7 and 8: L-rhamnose (750 µM). Line 9 and 10: L-rhamnose (1000 µM). Mw: prestained protein ladder V from Geneaid. Gels were stained with Comassie blue.

2. Substrate scope

2.1. Optimisation of the activity assays conditions

We started by determining the cofactor preference of each enzyme using NAD(P)H (0.2 mM) and the very active maleimide (20 mM) substrate in phosphate buffer (100 mM, pH 7.4) containing 20 % of DMSO at 25°C. While *Mc*OYE and *Tt*ENR were found to be NADPH-dependent, *He*OYE strongly preferred NADH and no activity was detected using the purified *Ph*ENR with both cofactors. Then, since the optimal pH for *Tt*ENR and *Ph*ENR has been reported previously, we investigated the optimal pH for *Mc*OYE and *He*OYE. citrate buffer (100 mM, pH 5.4), phosphate buffer (100 mM, pH 7.4), or bicarbonate buffer (100 mM, pH 9.4) containing 20 % of DMSO were tested (**Figure S4A**). While *He*OYE showed an optimal pH of 7.4 with 30 % and 75 % of the optimal activity at pHs 5.4 and 9.4, respectively, *Mc*OYE showed similar activities at pHs 5.4 and 7.4, with considerably reduced activity at pH 9.4. pH 7.4 was therefore selected for all tests. Finally, we determined the optimal temperature for each ER (**Figure S4A** and **B**). *Mc*OYE and *He*OYE displayed higher activities at 30 °C with reduced activities at higher temperature, the thermophilic *Tt*ENR exhibited maximum activity at 75 °C.

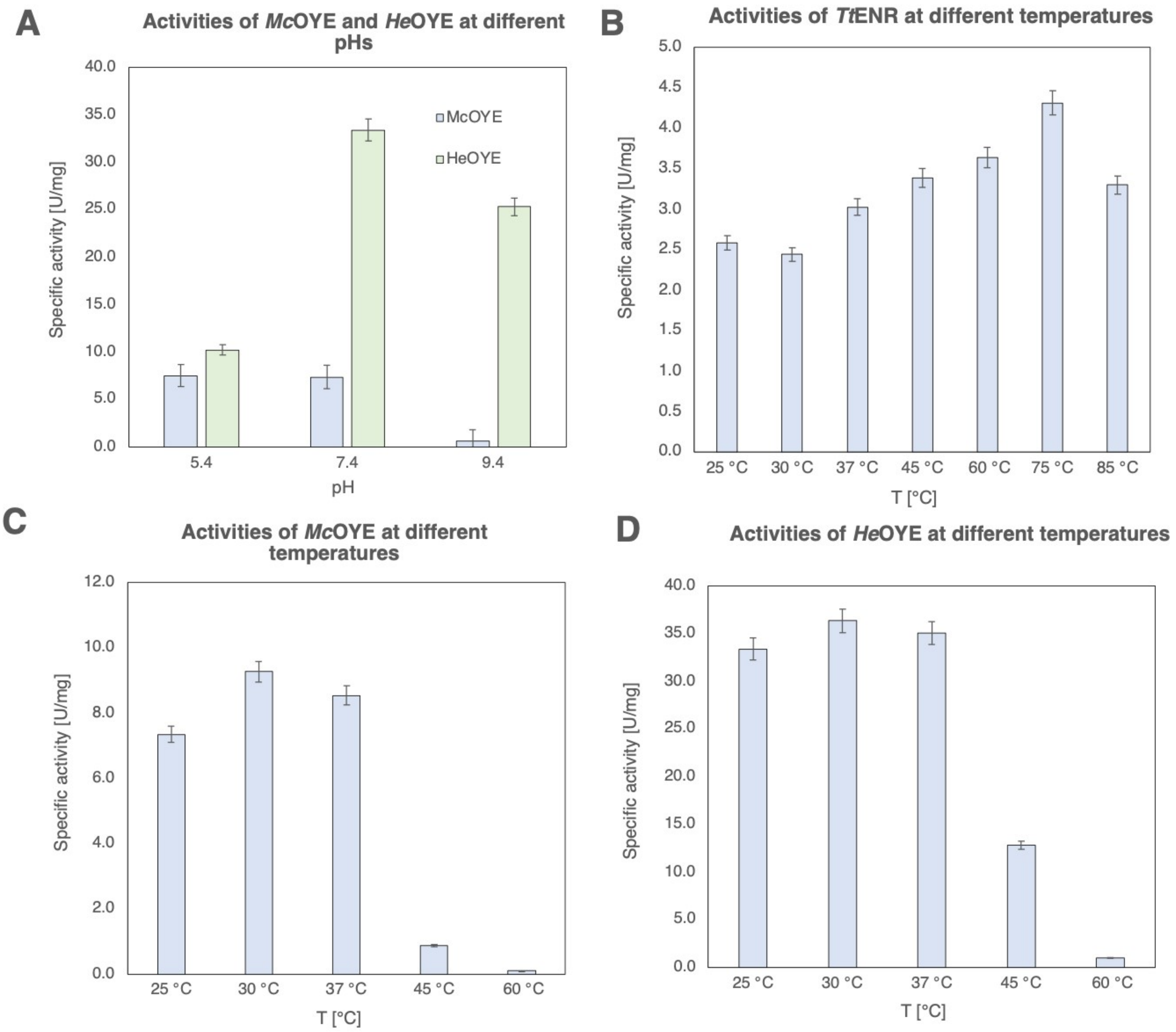

Figure S4: Investigation of the optimal conditions for the activity of ene-reductases using NAD(P)H (0.2 mM) and maleimide (20 mM). (**A**) *Mc*OYE and *He*OYE activities in citrate buffer (100 mM, pH 5.4), phosphate buffer (100 mM, pH 7.4), or in bicarbonate buffer (100 mM, pH 9.4) containing 20 % of DMSO at 25 °C. (**B**) *Tt*ENR, (**C**) *Mc*OYE and (**D**) *He*OYE activities in 100 mM phosphate buffer (100 mM, pH 7.4) at different temperatures. All experiment were done in triplicates.

3. Bioinformatics and immobilised enzyme development

3.1. Bioinformatic analysis

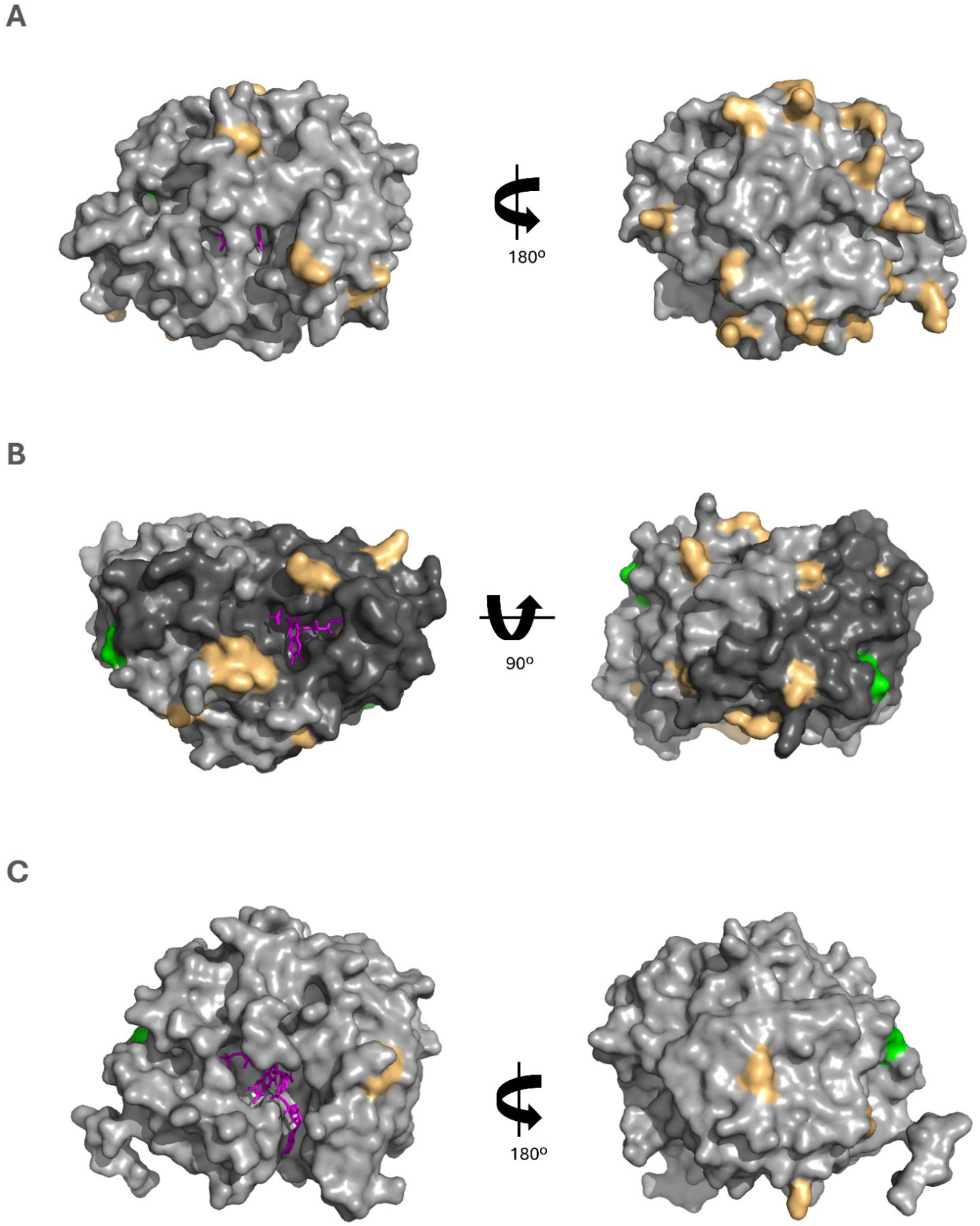

Figure S5: Lysine surface coverage and N-terminal for (**A**) *Mc*OYE (AlphaFold model, UniProt: S2JUG8) (**B**) *Tt*ENR (AlphaFold model, UniProt: Q5SH72), (**C**) *He*OYE (AlphaFold model, UniProt: E1V5B8). Lysine residues are coloured yellow, and the N-terminal green. The FMN cofactor in the active site is shown purple.

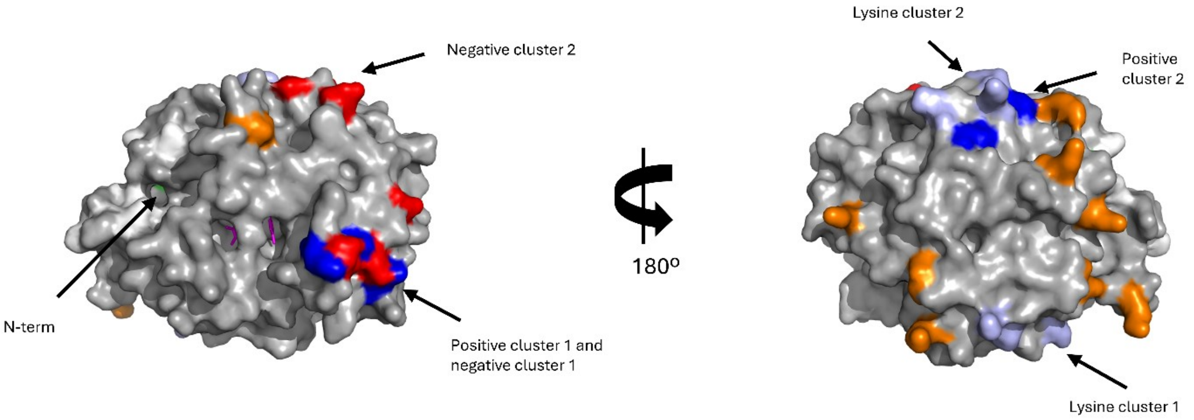

Cluster name	Residues
Positive 1	K146, K166, H173
Positive 2	K289, H291, R322, K323
Negative 1	D160, D1725, E174
Negative 2	D274, E277, E278
Lysine 1	K78, K120, K121
Lysine 2	K289, K323, K326

Figure S6: Cluster analysis of *Mc*OYE. Negative clusters are shown red, positive blue and lysine clusters light blue. Exposed lysines are shown in light orange. The residues involved in the formation of each cluster are detailed in the table below.

3.2. Immobilisation screening

Table S4: Immobilisation screening of *Mc*OYE

Entry	Support	Chemistry	Protein loading (mg/g)	Immobilization yield (%)	Recovered activity (%)	Expressed activity (U/g)
1	6BCL	Epoxy-Co	5	61 %	17.80 %	5.92
2	S2200*	Epoxy-Co	5	88 %	10.20 %	4.91
3	EC-HFA/S	Epoxy-Co	5	23 %	15.80 %	1.98
4	HFA403/S	Epoxy-Co	5	46 %	57.00 %	11.1
5	HFA403/S	Epoxy-Co	1	> 99 %	75.00 %	6.6
6	EP400/S	Epoxy-Co	5	37 %	3.40 %	0.68
7	EP403/S	Epoxy-Co	5	26 %	10.80 %	1.53

*Biocatalyst reusability compromised because of abrasion of the material.

Table S5: Immobilisation screening of *Tt*ENR

Entry	Support	Chemistry	Protein loading (mg/g)	Immobilization yield (%)	Recovered activity (%)	Expressed activity (U/g)
1	6BCL	Epoxy-Co	5	98 %	49.0 %	3.12
2	6BCL	Epoxy-Co	1	> 99 %	> 99 %	2.58
3	S2200*	Epoxy-Co	5	98 %	37.7 %	2.4
4	EC-HFA/S	Epoxy-Co	5	67 %	32.9 %	1.78
5	HFA403/S	Epoxy-Co	5	60 %	78.0 %	3.04
6	EP400/S	Epoxy-Co	5	91 %	45.1 %	2.67
7	EP403/S	Epoxy-Co	5	52 %	56.4 %	1.91

*Biocatalyst reusability compromised because of abrasion of the material.

Table S6: Immobilisation screening of *He*OYE

Entry	Support	Chemistry	Protein loading (mg/g)	Immobilization yield (%)	Recovered activity (%)	Expressed activity (U/g)
1	6BCL	Epoxy-Co	5	69 %	7.80 %	6.73
2	S2200	Epoxy-Co	5	82 %	4.70 %	4.87
3	EC-HFA/S	Epoxy-Co	5	91%	7.70 %	8.82
4	HFA403/S	Epoxy-Co	5	59 %	4.40 %	3.28
5	EP400/S	Epoxy-Co	5	60 %	7.20 %	5.46
6	EP403/S	Epoxy-Co	5	17 %	24.10 %	5.14
7	6BCL	Aldehyde	5	60 %	3.10 %	2.56
8	S2200*	Aldehyde	5	27 %	45.00 %	21
9	EC-HFA/S	Aldehyde	5	80 %	2.70 %	2.99
10	HFA403/S	Aldehyde	5	87 %	2.30 %	2.68
11	EP400/S	Aldehyde	5	91 %	2.50 %	2.95
12	EP403/S	Aldehyde	5	91 %	2.70 %	3.13
13	6BCL	Epoxy-Co	1	69 %	22.60 %	5.1
14	EC-HFA/S	Epoxy-Co	1	87 %	20.90 %	4.6
15	EP400/S	Epoxy-Co	1	98 %	4.50 %	1.1
16	EP403/S	Epoxy-Co	1	60 %	26.60 %	3.9
17	S2200*	Aldehyde	1	71 %	67.50 %	17
18	6BCL	Epoxy-Co	1	69 %	22.60 %	5.1
19	EC-HFA/S	Epoxy-Co	1	87 %	20.90 %	4.6
20	EP400/S	Epoxy-Co	1	98 %	4.50 %	1.1

*Biocatalyst reusability compromised because of abrasion of the material

4. Flow biocatalytic synthesis of 3-proprionaldehyde

4.1. Initial screening with free and immobilised enzymes at the 1 mM scale

We started by performing batch reactions using the free enzymes and those which InSEIT immobilised at the 1 mM scale, using NAD(P)H (2 mM) without cofactor recycling system in phosphate buffer (100 mM, pH 7.4) using 20 % of DMSO to solubilise cinnamaldehyde (**Figure S7**). In all cases, excellent conversions (89-100 %) were reached after 2 hours, except for *Tt*ENR/immobilised *Mc*OYE and the immobilised TtENR, for which steady state was reached after 18 and 24 hours, respectively.

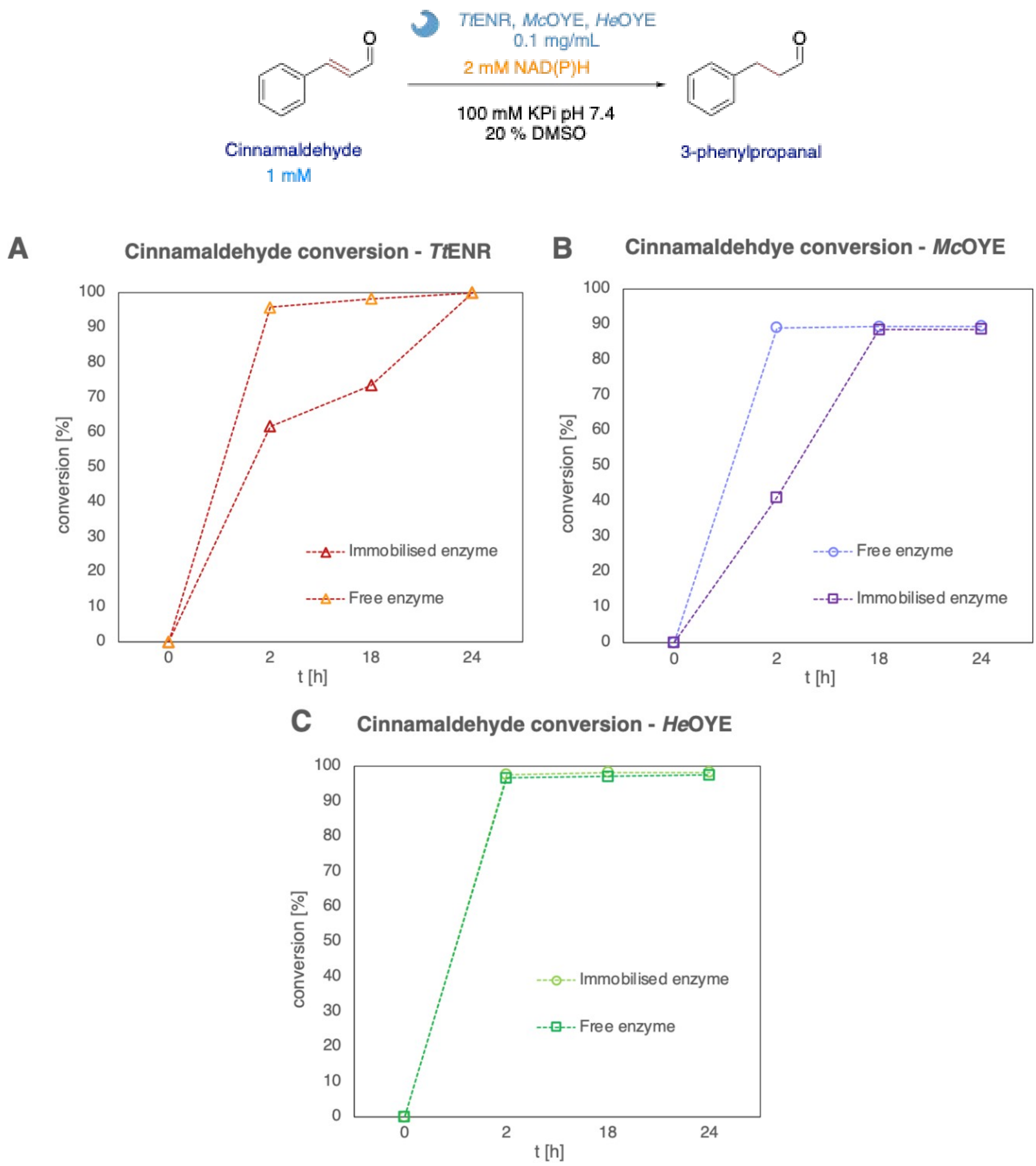

Figure S7: ER-mediated reductions of cinnamaldehyde in batch at the 1 mM scale using NAD(P)H (2 mM) without cofactor recycling system in 100 mM phosphate buffer (100 mM, pH 7.4) containing 20 % of DMSO with free and immobilised (**A**) *Tt*ENR, (**B**) *Mc*OYE, (**C**) *He*OYE. 0.1 mg/mL of the free enzymes was used, and the amount of resin corresponding to the same activity was added when using immobilised enzymes.

Reactions were performed at each enzyme's optimal working temperature: 30 °C for *Mc*OYE and *He*OYE, and 75 °C for *Tt*ENR.

4.2. Batch and flow biotransformations at the 10 mM scale with cofactor recycling system

We then performed flow biocatalytic transformations using this enzyme co-packed with immobilised glucose dehydrogenase from *Bacillus megaterium* (*Bm*GDH) using a 2 units of *He*OYE:1 unit of *Bm*GHD ratio, glucose (40 mM) and 0.1 equivalents of NAD(P)H (**Figure S8**).

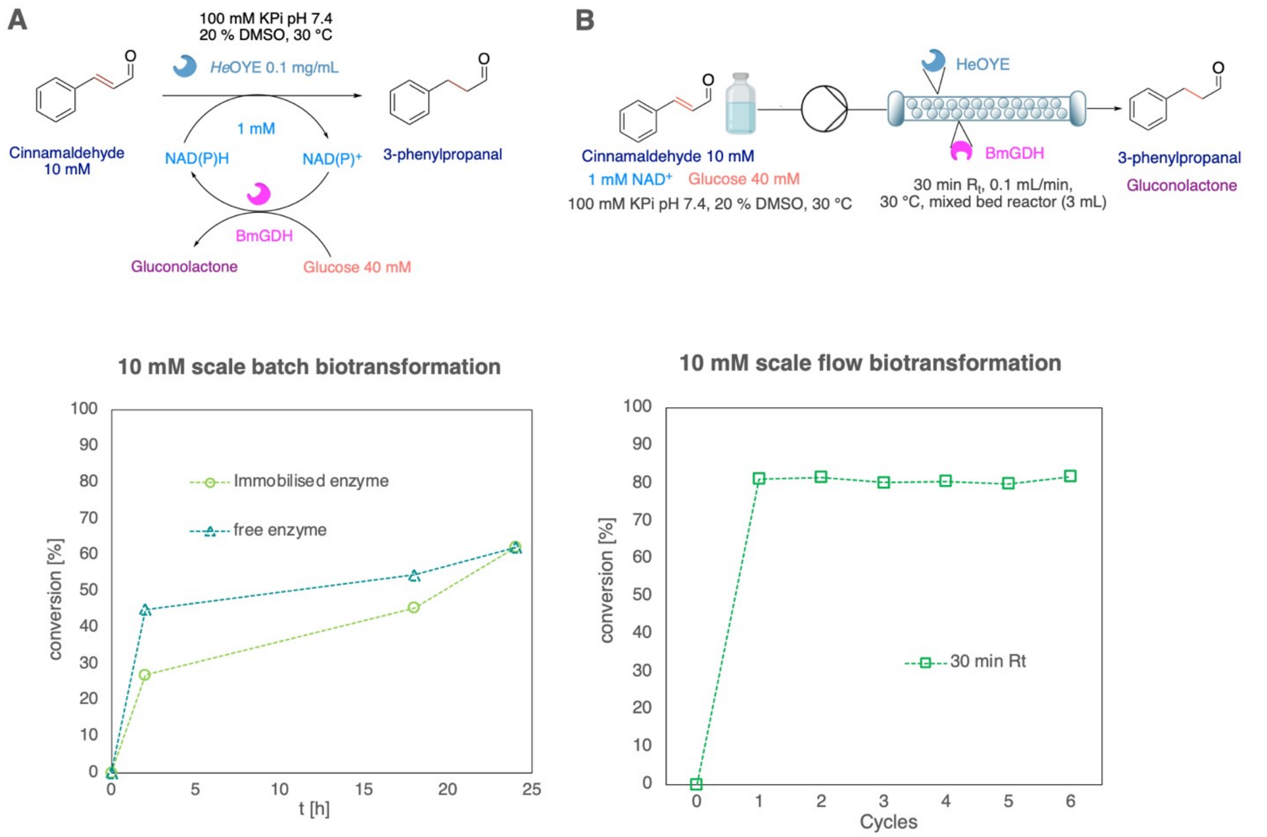

Figure S8: *He*OYE-mediated reductions of cinnamaldehyde at the 10 mM scale using NAD(P)H (1 mM) with *Bm*GDH as recycling enzyme partner and glucose (40 mM) as sacrificial substrate in phosphate buffer (100 mM, pH 7.4) containing 20 % of DMSO at 30 °C (**A**) in batch using 0.1 mg/mL of the free enzyme and the corresponding units of immobilised one. (**B**) in flow using 30 minutes of residence time in a 3 mL mixed bed reactor (0.1 mL/min flow rate). A cycle is defined as 30 minutes of reaction, *i.e* one residence time.

5. Sustainable indigo dyeing

5.1. UPO-mediated synthesis of indigo and 2-oxindole from indole

Optimisation of the H_2O_2 equivalents

The mechanism of indigo synthesis starting from indole proceeds via the epoxidation of the double bond of the 5-membered ring of indole leading to the corresponding 2-3-epoxide, followed by epoxide opening in two different ways, leading to indole hydroxylated at the 2 or 3 positions, which finally results in the spontaneous formation of 2-oxindole or indigo (upon air oxidation), respectively (**Figure S9**). We performed

the reaction at different scales using several equivalents of H_2O_2 in phosphate buffer (100 mM, pH 7.4) containing 10 % of acetonitrile (**Table S7**). A brownish colour was observed when higher equivalents of H_2O_2 were used, most probably indicating the presence of side products, as reported by Ullrich *et al.*[1] (**Figure S9A**). The presence of indigo was confirmed by the perfect match between the absorption spectra of the formed product compared with the indigo standard (**Figure S9B**). Almost complete indole depletion was observed in all cases, and the reaction was complete after 2 hours at all scales, with a representative example of the evolution of conversion as a function of time shown in **Figure S9C**. When reactions were performed at the 50 mM scale, H_2O_2 was added sequentially, as H_2O_2-driven heme destruction has been reported to impair UPO catalysis, especially with concentrations higher than 10 mM (**Figure S9D**).[5] Less than 6.5 % of indole degradation was observed in the CTRLs at scales below 10 mM, whereas 12.5 % indole degradation was detected at the 50 mM scale.

As noticed in **Figure S8C** and **D**, the main product of the reaction is not indigo but 2-oxindole. We investigated the effect of H_2O_2 equivalents on indigo conversion, as this was never performed in detail before (**Table S7**). In almost all cases, 64-85 % conversion 2-oxindole with less than 5 % conversion indigo were observed. While the 1 mM scale reaction using 5 eq. H_2O_2 resulted in 23.4 % conversion of 2-oxindole, indigo conversion was even lower than for other cases (0.7 %, **Table S7** entry 4). We also performed the reaction at pH 4.4 (in 100 mM citrate buffer) using 1.2 equivalents of H_2O_2 instead of pH 7.4, but results were very similar (68.5 % 2-oxindole and 5.1 % indigo conversion). Therefore, neither the equivalents of H_2O_2, the pH nor the reaction scale resulted in any improvement of indigo conversion.

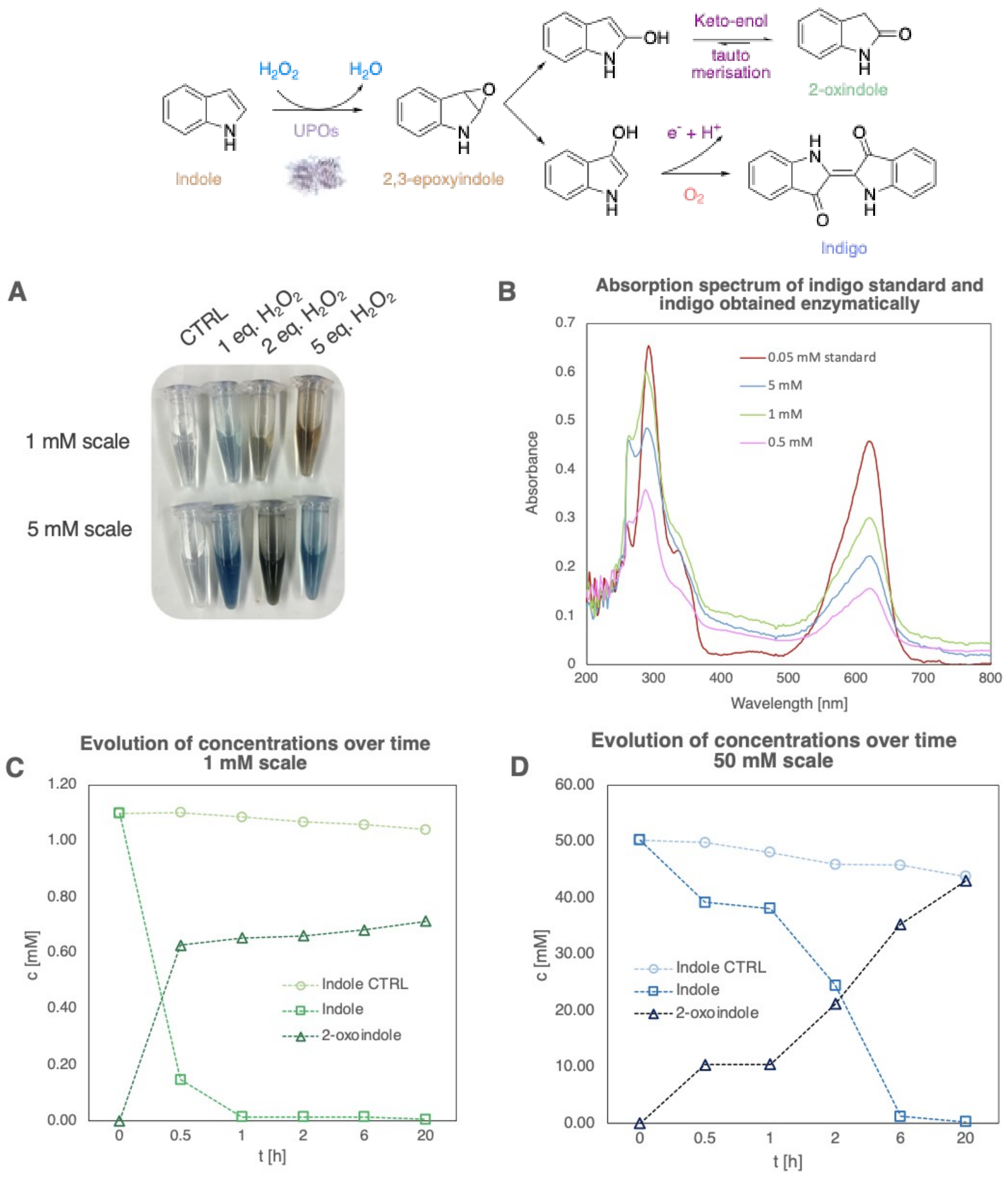

Figure S9: Biotransformations of indole into indigo at the 1 and 5 mM scales using different equivalents of H_2O_2 after 2 hours. (**B**) Analysis of indigo conversion by absorbance after its precipitation and centrifugation from the reaction mixture after 2 hours. (**C**) Biotransformation of indole into indigo at the 1 mM scale using 1.2 equivalents of H_2O_2. (**D**) Biotransformation of indole into indigo at the 50 mM scale using 1.2 total equivalents of H_2O2, added sequentially in 5 portions (0.24 eq. each hour). Reactions were performed in 100 mM phosphate buffer pH 7.4 containing 10 % of acetonitrile, in the presence of 1-50 mM indole, 1-5 eq. of H_2O_2, and 0.05 mg/mL of rAaeUPO (3U). Controls were run in the presence of all reaction components, except the enzyme. 2% spontaneous degradation of indole was detected at the 1 mM scale in the CTRL, as well as 12.5 % at the 50 mM scale.

Table S7: Synthesis of indigo from indole at different scales using different equivalents of H_2O_2. Reactions were performed in 100 mM phosphate buffer pH 7.4 containing 10 % of acetonitrile, in the presence of 0.1-50 mM indole, 1-5 eq. of H_2O_2, and 0.05 mg/mL of *rAae*UPO (3U). Reactions were monitored for 2 hours. A control was run in the presence of all reaction components, except the enzyme, showed 2-6.5 % spontaneous degradation of indole at the 1-10 mM scale, as well as 12.5 % at the 50 mM scale.

Entry	Scale [mM]	H_2O_2 equivalents	Indole depletion [%[	2-oxindole formation [%]	Indigo formation [%]
1	1	1	100	73.1 ± 3.7	2.3
2		1.2	100	64.5 ± 1.7	3.4
3		2	100	50.3 ± 5.7	1.8
4		5	100	23.4 ± 20.7	0.7
5	5	1	100	69.7 ± 4.1	5.5
6		1.2	100	83.8 ± 1.7	3.5
7		2	100	42.6 ± 1.3	4.2
8		5	100	68.3 ± 1.1	4.4
9	10	1	100	73.6 ± 2.3	4.9
10		1.2	100	76.2 ± 5.3	3.2
11		2	100	66.3 ± 3.4	5.1
12		5	100	71.1 ± 5.0	3.5
13	0.1	1.2	95.8	78.0	3.4
14	0.5	1.2	99.3	62.8 ± 3.8	3.6
15	50	1.2 (5x)	99.4	85.6 ± 1.1	3.5

Screening of another oxidant

Yamamoto *et al.*[7] published a practical one-pot chemical synthesis of indigo from indole *via* 3-position selective oxidation using a molybdenum complex as catalyst and 2.2 equivalents of cumene hydroperoxide in tert-butyl-alcohol. After optimisation of different metal catalysts (Mo, Ti, Ru), organic solvents, oxidants and reaction parameters (temperature, catalyst loading, reaction time), they obtained pure indigo in 81 % yield. Interestingly, they observed lower indigo conversion using H_2O_2 than cumene hydroperoxide. We therefore replaced H_2O_2 by cumene hydroperoxide in our reaction setup in an attempt to tune the selectivity of epoxide opening, and performed the reaction at pH 4.4 and 7.4 since cumene hydroperoxide was never used before in combination with UPOs (**Table S8**). However, the system performed worse than with H_2O_2, as less than 100 % of indole depletion was observed at scales higher than 1 mM, and the major reaction product was 2-oxindole with very low indigo conversions.

Table S8: Synthesis of indigo from indole at different scales using cumene hydroperoxide as oxidant. Reactions were performed in 100 mM citrate buffer pH 4.4 or in 100 mM phosphate buffer pH 7.4 containing 10 % of acetonitrile, in the presence of 1-10 mM indole, 1.2 eq. of H_2O_2, and 0.05 mg/mL of *rAae*UPO (3U). Reactions were monitored after 20 hours. A control was run in the presence of all reaction components, except the enzyme. 4 % spontaneous degradation of indole were detected at the 10 mM scale in the CTRL.

Entry	pH	Scale [mM]	Indole depletion [%[	2-oxindole formation [%]	Indigo formation [%]
1	4.4	1	100	82.3	2.3
2		5	100	89.1	1.5
3		10	69.1	56.6	0.9
4	7.4	1	100	81.6	2.5
5		5	87.9	85.2	1.2
6		10	55.1	47.1	0.7

5.2. ER-mediated reduction of indigo into leuco-indigo

Activity assays

We performed activity assay as previously described using indigo and indigo-carmine, a more soluble version of indigo. While other miscible solvents such as ethanol of methanol were partially able to dissolve indigo, DMSO was the best solvent to obtain a maximum concentration of 1 mM in the final activity assay. While no activity was detected in the control in the absence of indigo, small activity was detected using *Mc*OYE, *He*OYE and *Tt*ENR (**Table S9**, entries 1-3). Higher activity was detected with indigo carmine (**Table S9** entries 4-6), thus indicating that the main limitation for activity remains solubility. Activities measured using 0.1 mM of substrates are lower (**Table S9** entries 1 and 4), most probably indicating a K_M > 0.1 mM.

Table S9: Specific activities of the different ERs towards indigo and indigo carmine. Activity assays were conducted in the presence of 0.2 mM NAD(P)H, 1 mM of the respective substrates, in 100 mM phosphate buffer pH 7.4 containing 20 % of DMSO.

Entry	Substrate	$C_{substrate}$ [mM]	A_{McOYE} [U/mg]	A_{HeOYE} [U/mg]	A_{TtENR} [U/mg]
1	Indigo 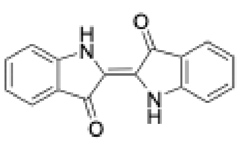	0.1	0.1 ± 0.1	0.1 ± 0.1	0.1 ± 0.1
2		0.5	0.3 ± 0.1	0.2 ± 0.1	0.2 ± 0.1
3		1	0.2 ± 0.1	0.2 ± 0.1	0.1 ± 0.1
1	Indigo carmine 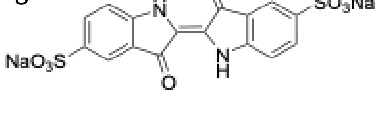	1	0.9 ± 0.3	1.3 ± 0.1	0.7 ± 0.4
2		1	1.5 ± 0.3	2.3 ± 0.1	1.4 ± 0.4
3		1	1.2 ± 0.3	2.5 ± 0.1	1.1 ± 0.4

Comparison of chemical and ER-mediated dyeing

In the absence of better analytical methods, we dyed cotton cloth chemically at pH 12 using 40 mM of sodium dithionite ($Na_2S_2O_4$) or glucose as reducing agents. Using indigo, only the chemical reduction at basic pH resulted in dyeing, with $Na_2S_2O_4$ producing the yellow leuco-indigo after 10 minutes only, while an overnight incubation was required using glucose (**Figure S10A** and **Table S10** entries 1-6). When the more soluble indigo carmine was used instead, reduction into the soluble yellow form was observed in all cases after 2 hours, although with a slightly different shade of yellow for the chemical reductions (**Figure S10B**). Overnight incubation revealed that this colouration was actually due to dye degradation in the case of the chemical reductions (**Table S10** entries 7-9), whereas the system stayed yellow with the ER-mediated reactions, even after 1 week (**Figure S10C** and **Table S10** entries 10-12).

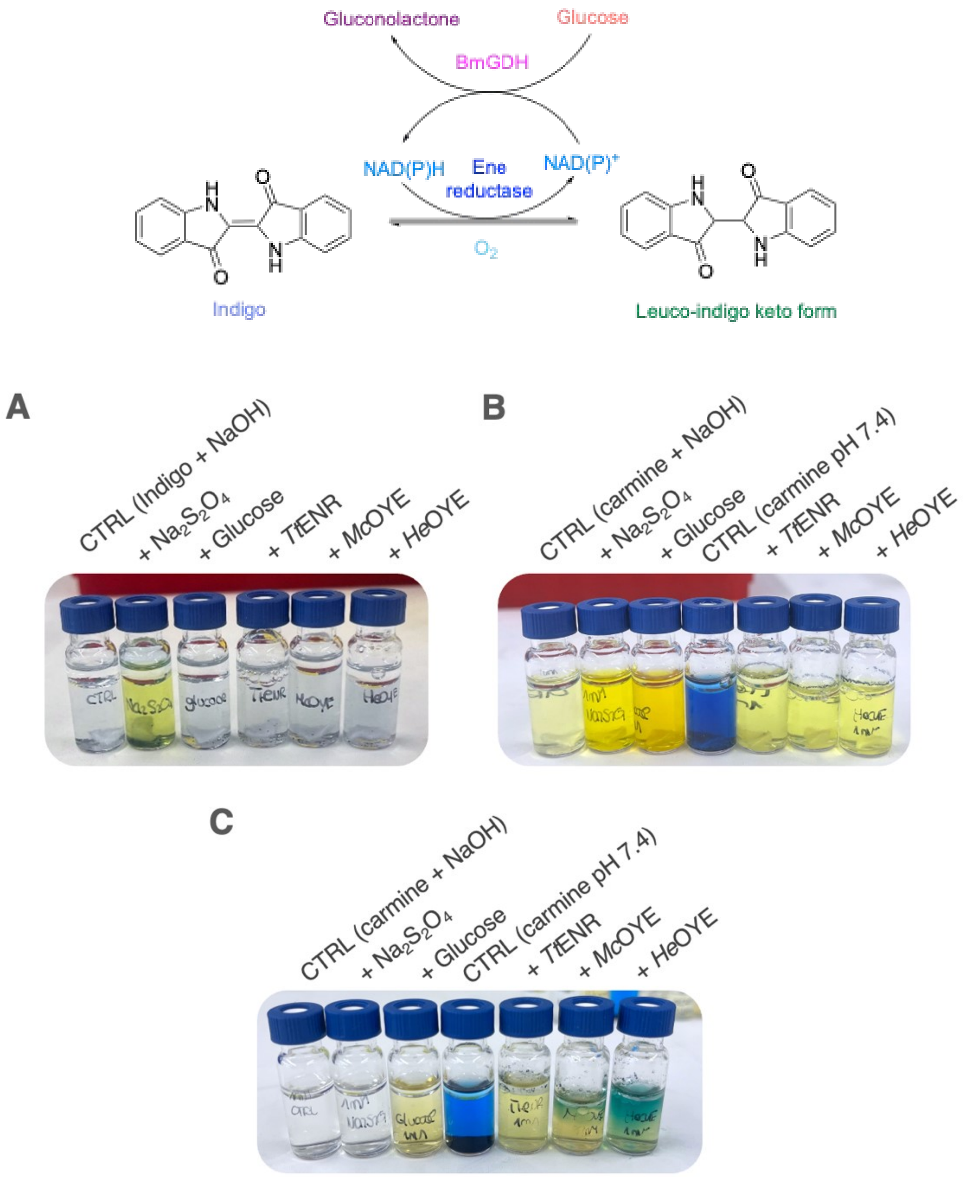

Figure S10: Chemical and enzymatic dyeing of (**A**) indigo (1 mM) after 2 hours, (**B**) indigo carmine (1 mM) after 2 hours, (**C**) indigo carmine (1 mM) after 1 week.

Table S10: Chemical and enzymatic dyeing of 0.5 or 1 mM of indigo or indigo carmine. Chemical dyeing was performed at pH 12 in the presence of Na_2S2O_4 (40 mM) or glucose (40 mM), NaOH (200 mM), and enzymatic reactions were performed in the presence of 1 mg/mL of ene-reductase, 0.5 mg/mL of glucose dehydrogenase, glucose (40 mM) and NAD(P)H (1 mM). A layer of mineral oil was added on the top of each vial and solution were bubbled with N_2.

Entry	Type of reduction	Substrate	Reducing agent/enzyme	Recycling system	Observations
1	Chemical (pH 12)	Indigo	None (CTRL)	-	No dyeing
2			$Na_2S_2O_4$ (40 mM)		Reduction and dyeing after 10 minutes
3			Glucose (40 mM)		Reduction and dyeing overnight
4	Enzymatic (pH 7.4)		*Tt*ENR (1 mg/mL)	*Bm*GDH (0.5 mg/mL) + glucose (40 mM)	No dyeing
5			*Mc*OYE (1 mg/mL)		
6			*He*OYE (1 mg/mL)		
7	Chemical (pH 12)	Indigo carmine	None (CTRL)	-	Decolourisation but no dyeing
8			$Na_2S_2O_4$ (40 mM)		Decolourisation but no dyeing, red colour
9			Glucose (40 mM)		Decolourisation but no dyeing
10	Enzymatic (pH 7.4		*Tt*ENR (1 mg/mL)	*Bm*GDH (0.5 mg/mL) + glucose (40 mM)	Reduction and dyeing after 10 minutes
11			*Mc*OYE (1 mg/mL)		Reduction and dyeing after 10 minutes
12			*He*OYE (1 mg/mL)		Reduction and dyeing after 10 minutes

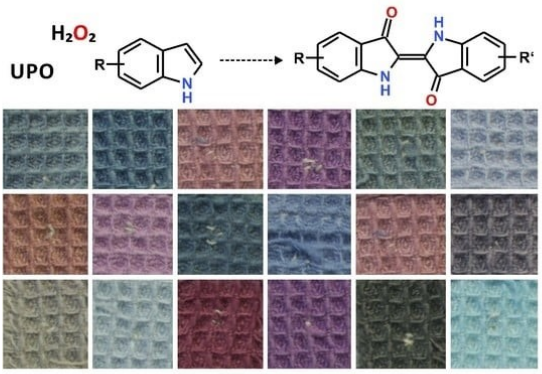

Figure S11: Graphical abstract of the synthesis of indigo dyes from indole derivatives by using unspecific peroxygenases and their application for in-situ dyeing.[6]

6. Supplementary references

1 G. Steinkellner, C. C. Gruber, T. Pavkov-Keller, A. Binter, K. Steiner, C. Winkler, A. Łyskowski, O.Schwamberger, M. Oberer, H. Schwab, K. Faber, P. MacHeroux, K. Gruber, *Nat Commun.,* 2014, **5**, 4150.

2 A. Romagnolo, F. Spina, A. Poli, S. Risso, B. Serito, M. Crotti, D. Monti, E. Brenna, L. Lanfranco, G. C. Varese, *Sci. Rep.,* 2017, **7**, 12093.

3 S. Sharma, Y. Monga, A. Gupta, S. Singh, *RSC. Adv.,* 2023, **13**(21), 14249–14267.

4 Expasy, "ProtParam", can be found under https://web.expasy.org/protparam/ (accessed 8 August 2025).

5 D. Roura Padrosa, V. Marchini, F. Paradisi, *Bioinformatics*, 2021, **37**(17), 2761–2762.

6 R. Ullrich, M. Poraj-Kobielska, O. M. Herold-Majumdar, J. Vind, M. Hofrichter, *Catalysts,* 2021, **11**(12), 1495.

7 Y. Yamamoto, Y. Inoue, U. Takaki, H. Suzuki, *Bull. Chem. Soc. Jpn.,* 2011, **84**, 82–89.
